# Model-Driven Methodology for Rapid Deployment of Smart Spaces Based on Resource-Oriented Architectures

**DOI:** 10.3390/s120709286

**Published:** 2012-07-06

**Authors:** Iván Corredor, Ana M. Bernardos, Josué Iglesias, José R. Casar

**Affiliations:** Data Processing and Simulation Group, School of Telecommunication Engineering, Universidad Politécnica de Madrid, Avda. Complutense 30, 28040 Madrid, Spain; E-Mails: abernardos@grpss.ssr.upm.es (A.M.B.); josue@grpss.ssr.upm.es (J.I.); jramon@grpss.ssr.upm.es (J.R.C.)

**Keywords:** smart space, Web of Things, Model Driven Architecture, ontology-driven architecture, UML profile, development methodology, resource-oriented architecture

## Abstract

Advances in electronics nowadays facilitate the design of smart spaces based on physical mash-ups of sensor and actuator devices. At the same time, software paradigms such as Internet of Things (IoT) and Web of Things (WoT) are motivating the creation of technology to support the development and deployment of web-enabled embedded sensor and actuator devices with two major objectives: (i) to integrate sensing and actuating functionalities into everyday objects, and (ii) to easily allow a diversity of devices to plug into the Internet. Currently, developers who are applying this Internet-oriented approach need to have solid understanding about specific platforms and web technologies. In order to alleviate this development process, this research proposes a Resource-Oriented and Ontology-Driven Development (ROOD) methodology based on the Model Driven Architecture (MDA). This methodology aims at enabling the development of smart spaces through a set of modeling tools and semantic technologies that support the definition of the smart space and the automatic generation of code at hardware level. ROOD feasibility is demonstrated by building an adaptive health monitoring service for a Smart Gym.

## 1. Introduction

During the first decade of the 21st century, technologies for *pervasive computing* have evolved to make real Weiser's of *ubiquitous* and *calm computing* [1]. Weiser devised a world in which computers were fully integrated into everyday environments, transforming them into smart spaces [2] capable of seamlessly providing adaptive services to be consumed in a natural and non-intrusive way.

The generalization of these smart spaces may have a beneficial impact on society and economy, serving as basis for applications in different domains, such as sustainability, support to daily living or personal health. For example, a United Nations report [3] estimates that the urban population will grow by 2.3 billion over the next 40 years, thus around 70% of the World's population will live in cities by 2050. The increased industrialization and consumption related to the growth of the cities may generate adverse transformations of the natural environment such as greenhouse effects or exhaustion of natural resources. Smart spaces will help to achieve more sustainable cities, particularly with respect to the management of energy resources, at the same time that they enable optimal food and goods production (e.g., smart greenhouses, smart factories) and traceability from the seed to the plate.

Among the many technological challenges involved in the evolution of smart spaces, the roadmap for Micro-Electro-Mechanical (MEMS) technologies is especially relevant; in the last decades, they have evolved towards a wide and inexpensive catalogue of small nodes equipped with wireless interfaces as well as sensor and actuator devices, although they are still far away from the ideal *Smart Dust* paradigm [4]. In any case, this on-going electronic revolution has contributed to create a solid technological base for the development of augmented daily objects, which go beyond their standard capabilities thanks to their bundled sensors, actuators and interfaces (e.g., touch screens, keyboards, microphones, *etc.*). In particular, in this paper we refer to smart object as “*a computationally augmented tangible object*”, that is “aware” of its own situation as participant of an ecosystem (*i.e.*, a smart space). This “minimum intelligent unit” is characterized by a set of behaviors, communication capabilities and interaction methods with peers and real-world entities, and may play many different roles within those smart spaces, performing individual or collective objectives.

Together with the increasing availability of sensor and actuator embedded devices, novel software paradigms to facilitate the integration of these devices in collaborative environments are handling both “horizontal” and “vertical” planes. The “horizontal” plane is characterized by Machine-to-Machine (M2M) interactions that refer to the communication between devices’ functionalities. These M2M interactions are being supported by a recently research field that tries to solve the communication according to IP-based networks, that is, the Internet of Things (IoT) [5]. The IoT paradigm suggests an IP-based internetworking schema in order to get resource-constrained devices ready to be plugged into Internet. From the pillars built by IoT research field, the concept of the Web of Things (WoT) [6] has arisen with the promise of bringing those interconnected infrastructures of embedded devices into the Web services cloud. The WoT establishes the “vertical” plane of interaction allowing devices to work collaboratively, even among those belonging to different networks or systems. That communication is performed directly by the underlying devices through gateway-based systems that hide the heterogeneity of technologies.

Nowadays, both IoT and WoT concepts are being developed under researches in diverse fields (e.g., communication protocols, semantic technologies or reasoning engines) with the final purpose of developing technology to allow isolated “islands” of sensor and actuator devices to be part of a connected network of heterogeneous smart spaces. In a future, smart spaces composed of hundreds, even thousands, of Web-enabled smart objects, will become omnipresent entities providing *real-world* services. Thus next steps require developing sound methodologies to improve some links in the development chain of IoT and WoT and to optimize the cost of spreading smart spaces. In particular, there is still a need for technologies that enable the deployment of *real-world* services by abstracting the specificities of the enabling devices: up to now, any developer who wants to address the deployment of a smart space still needs to have strong knowledge on general and specific technology aspects as communication protocols (e.g., IEEE 802.15.4, 6LoWPAN, uIP, uPnP…), platforms of wireless sensor and actuator networks (WSANs), specific programming languages, service discovery mechanisms, or security procedures, among others.

Some Internet-oriented approaches facing the problem of facilitating device-layer abstraction include the use of Web Services (WS-*) based on Service-Oriented Architectures (SOA). This orientation is often used for stand-alone infrastructure-based designs, but it reduces its performance and functionalities when applied to resource-constrained devices (e.g., mobile devices [7]). Newer trends [8–11] are taking up again the concept of *resource* that was defined at the beginning of the 2000s in [12], supported by the REpresentational State Transfer (REST) architectural style, in order to build lightweight Resource-Oriented Architectures (ROA) that are more suitable to integrate and deploy in constrained embedded devices as studied in previous works [13,14].

The ROA client-server design principles are characterized by their simplicity and versatility due to its technological base, highlighting addressability of resource through the Universal Resources Identifier (URI) scheme, and Hypertext Transfer Protocol (HTTP) to define methods to access and interact with resources. Despite its promising characteristics, existing platforms do not still fully decouple the device layer from the service layer.

For this reason, some research works are addressing domain-specific development frameworks based on patterns and models since they can reduce costs and deployment time and increase scalability in large deployments of smart spaces. A recent trend on this field is betting on the Model Driven Engineering (MDE) principles [15–17] with the aim of facilitating the management and organization of service areas. By using MDE-based methodologies, managers, analysts and developers may increase their productivity since it simplifies the process of design and development by using models and design patterns. Additionally, those approaches can increase the communication between participants working on the system development via standardization of languages and terminology, e.g., by means of a domain specific language designed by a company in order to model agent life-cycle for a software product. Furthermore, MDE-based approaches fit very well with the IoT and WoT paradigms as they facilitate:

- abstracting every part of the system through high-level models independently of the underlying software and hardware technologies.
- decoupling consumers and providers of contextual resources (sensor, actuators and logic processes), enabling a real reuse of model artifacts and software components.
- providing a modeling framework to facilitate rapid and agile prototyping of complex deployments even for non-expert developers.

The research presented in this article takes advantage of the MDE principles to build a holistic development methodology involving a common, semantically expressive abstraction model, to specify a smart space with its specific services. The initial motivation was to provide a versatile solution to facilitate the development of different smart spaces service scenarios, to be composed of heterogeneous sensors, actuators and logic processors interacting among them through a variety of mechanisms. From that ambitious point of view, we propose the Resource-Oriented and Ontology-Driven Development (ROOD) methodology, which improves traditional MDE-based tools through semantic technologies for rapid prototyping of smart spaces according to the IoT and WoT paradigms. With the aim of providing expressivity to the ROOD's models, we have designed a Unified Modeling Language (UML) profile [18], so called the Smart Space Modeling Language (SsML), that defines a Domain Specific Model (DSL) including singularities of smart spaces, *i.e.*, *interactions*, *participants*, *resources*, and *platforms*. The ROOD methodology involves two models that are instances of the SsML: the *Smart Object Model* (SOM) and the *Environment Context Model* (ECM). The ECM is focused on describing high-level behaviors, interactions and context information of the entire smart space. On the other hand, the SOM model defines the processing aspects related to the sensing and actuating capabilities of the smart objects, as well as the context model they manage; moreover, SOM models encapsulate these concepts into RESTful resources [12]. Both models comply with the specific viewpoints of the system, which were designed to verify the use of their elements. ROOD methodology also exploits semantic technologies in order to verify the integrity of the deployment scenarios used by the models. Each scenario would be described in Knowledge Bases (KBs) instantiated accordingly. The *Smart Space Ontology* (SSO) includes every concept needed to conceptualize a smart space populated of smart objects according to their sensing and actuating capabilities, that are offered through RESTful interfaces.

The rest of the paper is structured as follows. Section 2 gathers relevant works on approaches for modeling services and resources in IoT and WoT. Section 3 presents major research challenges analyzed from related work and how they are tackled by our proposal. Section 4 describes an overview of the major features of the ROOD methodology, focusing on its design principles. Section 5 presents the guidelines of ROOD methodology along its different stages. Section 6 describes first results from an early implementation of ROOD based on a Smart Gym deployed on a Smart Hotel. Section 7 concludes the paper with future work.

## 2. Related Work and Background

The analysis of the state of the art of development methodologies shows some interesting proposals providing applicable ideas to construct a holistic methodology for smart spaces. However, to the best of our knowledge, no one addresses the development of specific Model-Driven methodologies to support the design and deployment of smart spaces by modeling from the scenario context to its business logic, and ready to be subsequently deployed on a ROA. Additionally, with regard to semantics, few authors have integrated ontological technologies to support model transformations or validations in Model-Driven approaches, even if there is an increasing interest on the topic. Trends that integrate ontologies with Software Engineering (e.g., Ontology Driven Software Engineering or Ontology Driven Architectures) (e.g., [19], [20] or [21]) have demonstrated how the use of ontologies can enhance the development process within Model Driven methodologies of pervasive services, although they introduce some burden coming from the required effort for the ontology definition and its management.

As it was described in the previous Section, our proposal needs to consider synergies among different technological and research topics. This way, we have organized the following review of the *state of the art* around three specific issues that are relevant for the proposal: (i) MDE-based methodologies for integration with service oriented architectures, semantic models, and model transformation; (ii) ontology-driven approaches for software engineering and semantic approaches to model smart environments; and (iii) Resource-Oriented approaches to develop and deploy REST resources within the WoT paradigm.

### 2.1. MDE-Based Approaches

Model-Driven Engineering (MDE) [22] is a generic software engineering methodology that uses abstractions (models) as the main artifacts during the development process. In short, the major benefits achieved by MDE are related to enhancing productivity, reusability, portability, maintainability and interoperability. In the field of pervasive computing, MDE allows developing and deploying software projects without having previous knowledge about specific programming languages or platform technologies. The Model Driven Architecture (MDA) [23] is a MDE-based initiative founded by the Object Management Group (OMG), that proposes an *open and vendor-neutral approach* to tackle complex business systems. The MDA specification places emphasis on a layered process, using different viewpoints. In MDA, a *viewpoint* on a system is a technique that provides a way of representing functionalities of a system through interfaces and specific design patterns, which characterizes the behavior and business processes of any application deployed on a platform without concern for technical details. A *platform* is a system or set of subsystems in which software artifacts are launched on. MDA proposes three kinds of viewpoints stratified in three models: (i) Computational Independent Model (CIM), (ii) Platform Independent Model (PIM) and (iii) Platform Specific Model (PSM). These models have to be machine-readable so that they are successively transformed into code stubs, schemas, test harnesses, and deployment scripts for diverse platforms [22]. OMG provides standardized tools to perform the MDA development methodology, particularly the Unified Modeling Language (UML) [18]. Domain Specific Modeling Languages (DSML) can be designed by means of a profile mechanism, provided by UML 2, with enough expressiveness and precision for almost any technological domain.

The design of our SsML is inspired by some UML profiles supported by OMG. Some features of the SoaML (Service-oriented architecture Modeling Language) [24], which specifies a profile focused on designing services and entities participating in a SOA-based system, have been considered in the design of SsML to model both business participants and interactions among them. From those features, roles of participants can be defined; it is also feasible to define their collaboration rules within a pervasive architecture. Another UML profile to be considered when modeling embedded and real-time systems, is MARTE [25]. This is a very detailed profile that provides a foundation for modeling both hardware and software aspects for RTES (Real-Time Embedded System). Specifically, MARTE is focused on analyzing and modeling performance and scheduling of resources for RTES. MARTE provides a framework to carry out quantitative analysis (Quantitative Analysis Modeling profile). This part of MARTE provides tools to model Interaction Overview Diagrams (IOD), a specialized form of UML activity diagrams for describing the chain of actions needed to perform an activity. IOD can model the workload of an application, including *triggers* to express the stimulation of *responses* and *transactions* that corresponds to partition activities in UML. This characteristic of MARTE is very useful to model behavioral aspects of smart spaces, including actions to collaboratively carry out an objective.

Apart from stand-alone tools for modeling system artifacts, MDA's development principles have motivated some research works considering the whole development process, from CIM to PSM; these works involve meta-models and transformations between the three modeling stages. In [26] a model-driven Service-Oriented Development Method (SOD-M) is proposed as methodology to model service-oriented architectures. SOD-M specifies a CIM, which models the *business view* of the system, and a PIM, that models the *information system view*. SOD-M defines a complete methodological approach to define mappings with the aim of detecting errors and inconsistencies on models that allow supporting an enhanced alignment between CIM and PIM. Although this approach clearly defines the phases of a MDA methodology, it is not suitable for a WoT-based system since SOD-M is constrained to SOA for Web-based information systems that, as previously said, contravene some requirements of embedded environments such as lightness in information transactions.

Few MDA approaches are focused on improving model-driven development through semantic technologies for supporting heterogeneous deployment scenarios and platforms definition. The authors of [27] state that models and metamodels are representations of part of the reality. They suggest that ontologies can support construction of models of a system by reasoning about their consistency with regard to the reality. One of the first contributions in this line was the W3C's proposal called the Semantic Web Best Practices and Development (SWBPD), whose main contribution is an Ontology Driven Architecture [17]. Several research works have been performed over that early idea. For instance, [28] proposes an MDA-based methodology to reduce the burden when using ontologies for pervasive systems. Authors focus their research on a model transformation mechanism for the generation of code for context-aware applications. In order to define context for pervasive services, authors proposed a Context Ontology Model (COM) consisting of the Upper-Level Context Ontology Model (ULCOM) and the Extended Specific Context Ontology Model (ESCOM). Moreover, a Model Driven Integration Architecture (MDIA) is provided jointly with a transformation mechanism.

Katasonov *et al.* [16] propose an extension of MDA following the Ontology Driven Software Engineering (ODSE). This methodology employed ontologies in the place of CIM, to support the generation of parts of PIM, achieving some level of automation. Ontologies used in this methodology are classified within three groups: (i) Domain ontology (to define concepts of the application domain), (ii) Task ontology (to define domain operations) and (iii) Ontology of software (to define concepts used on software fields). This proposal also introduces a modeling tool (Smart Modeller) enabling developers of smart applications to graphically build a model and then automatically generate program code for a specific platform.

Walter *et al.* [29] present an approach which uses ontological resources in order to support a typical MDE methodology through semantic reasoning services supporting the different roles involved in the methodology: DSL designers and DSL users. Firstly, this approach integrates Ecore meta-metamodel and OWL metamodel at the M3-layer of MDA with the aim of providing for both DSL designers and DSL users. DSL designers can then design consistent DSLs due to new constraint analysis. On the other hand, formal model-theoretic semantics enable the implementation of reasoning services to help DSL users to permanently validate domain models in order to detect inconsistencies as well as to analyze them and to get assistance in the modeling process.

Other MDA-based approaches [30,31] take advantage of design patterns to facilitate reusability, accurate automation, and granularity between transformations. These approaches can be useful for smart spaces development since there are many entities, interactions and behaviors that take place in many different scenarios following similar patterns.

In line with MDA approaches, and closer to WoT field, Rauf *et al.* [32] propose an approach to model conceptual and behavioral aspects of RESTful services through UML diagrams. These contracts can generate a standard WADL document describing such interfaces. Similarly, Laitkorpi *et al.* [33] propose a Model-Driven Process addressing the production of RESTful services by means of several phases, including intermediate models and transformations, from behavioral to information models.

The Model-Driven approaches previously analyzed are generalist and their major foundations can be taken into account when designing new MDE-based approaches. However, regarding modeling of smart spaces, there are many drawbacks that need to be tackled through specific solutions, which solve semantic descriptions and mechanisms to achieve the integration of smart spaces into the WoT paradigm. These major issues are analyzed next.

### 2.2. Semantic Approaches to Model WoT-Based Smart Spaces

Several works support the use of semantic technologies to overcome the limitations of contemporary standard-based and embedded devices for smart spaces (e.g., [34], [35] or [19]), evolving from a syntactic and procedural interoperability (*i.e.*, regarding standards, data formats, protocols, *etc.*) to semantic interoperability.

In contrast to previous works, basically focused on semantic vocabularies for specific cases of smart spaces deployments, the model presented in [19] could be considered one of the most complete and generic ones; it includes ontologies to model smart objects, sensors, services and events. However, it does not consider domain ontologies, that allow adapting MDA-based models to a variety of scenarios by defining context-aware information.

One of the main advantages (and lei™otif) of semantic models is their ability to be reused and shared: to date, several works modeling smart spaces include standard (or well-known) ontological models. For instance, OWL-Time [36] and Geo-OWL [37] ontologies can be used to model time and location concepts, respectively; furthermore, device capabilities representation [38] can be modeled using Delivery Context Ontology [39], a formal model of the characteristics of the environment in which devices interact with other services.

It is important to highlight the results generated from standardization groups that are focused on semantically modeling specific concepts which may be applied to smart spaces (*i.e.*, systems, sensors, devices or services). In this research area, the Open Geospatial Consortium (OGC) is building momentum. The OGC founded the Sensor Web Enablement (SWE) initiative with the aim of designing a set of standards for the development of a geo-located and interoperable Sensor Web. Some of its results are already considered as standards, e.g., SensorML for describing processes within sensors and observation processing systems. A research work related to OGC's approach is the Semantic Sensor Network (SSN) ontology [21]. The SSN ontology is the result of the work performed by the W3C Semantic Sensor Network Incubator Group (SSN-XG). It provides a semantic framework to define networked devices and systems with sensor capabilities. This ontology is divided into modules, which allow reasoning about sensorial capabilities, origin of measurement and the interconnection of an undefined number of sensors in a macro system. One of the major objectives of the SSN-XG group was to improve the set of standards of OGC's SWE in order to align SSN with them (it uses SensorML definition for some of its modules); however the extensions to other concepts in the IoT and WoT are not included in this ontology. Other standardization groups are concerned on the declaration and specification of services. In this field, one of the most popular approaches is OWL-S [40]. This is an ontology-based model for service description: a functional description (inputs, outputs, conditions and effects) and a non-functional description (quality of service or classification). The service model paves the way towards automation when discovering, invoking, composing, and monitoring Web resources offering particular services. This ontology is focused on defining semantic Web services but they are not directly applicable to WoT approaches, where the RESTful architectural style has demonstrated to be more feasible. Anyways, OWL-S ontology is usually used as upper ontology to define business models that support resources exposed through the WoT paradigm as the proposed in this paper (see Section 4.4) or de Suparna *et al.* [41].

ROA-based approaches promote the appearance of semantics solutions for modeling different aspects of RESTful frameworks. A ROA-compatible ontology system is described in [42]. This approach includes four interrelated ontologies describing *resources*, *cooperation*, *domain* and *services*. A *Capability Injection* pattern allows managing the resources’ lifecycle by providing mechanisms for abstraction, classification and resources-oriented software architecting. Although this approach provides a service ontology, it does not define mechanisms to associate resources to the underlying services or business processes that support those resources. Another approach [41] addresses the creation of a semantic model for the provision of real-world services by means of RESTful frameworks. This work proposes a whole semantic model encompassing different data providers and data descriptor components with the aim of describing the entities, resources and services models that are involved in a WoT paradigm. This model support aspects such as the spatial and temporal context, as well as thematic data related to defined resources in line with the Linked Data paradigm. Moreover, this solution can be used to dynamically manage association between entities, spaces and resources. The resulted ontology is inspired on standard ontological resources, particularly SSN and OWL-S.

Other approaches assume the current SOA predominance over ROA and try to find solutions to evolve from one domain to other keeping in mind common points among *services* and *resources*. In order to evolve from a SOA to a ROA domain, Wei *et al.* [9] propose a Resource-oriented Information Supported Framework that, starting from an initial ontology and describing legacy enterprise elements, achieves a domain ontology based on resource model to meet RESTful services.

### 2.3. Resources-Oriented Frameworks Approaches

The growing interest in modeling REST-based resources [12] has led to many research projects concerning lightweight ROA. Prior to this work, we have proposed Service-Oriented Middleware for integrating embedded pervasive devices into the WoT [8,43]. These research works addressed the design and development of a middleware for wireless embedded devices. This middleware provides a framework to expose sensor and actuator capabilities as RESTful services through a gateway-based approach. Our research demonstrated the convenience of using ROA approaches for integrating embedded devices into Internet and, particularly, following WoT principles; recent trends point out to these kinds of solutions as the foundation for the future smart spaces.

In the line of our previous works, Christophe *et al.* [44] propose a framework which enables the integration of embedded devices (*objects*) into a WoT perspective. This framework specifies the semantics of connected objects supporting flexible configurations for different scenarios. This feature allows creating Web-enable objects by exposing their functionalities as RESTful services, as well as composing sets of objects in order to offer advanced services. They also provide an object browser whose major features consist of discovering objects and requesting associated services via PC or Smartphone.

Some other Resources-Oriented Frameworks are based on toolkits that facilitate the development and deployment of RESTful applications for the WoT. AutoWoT [45] aims at providing a rapid integration of smart devices into the Web by automatically generating both applications and server software components. The approach addressed in [11], gathers a resource semantic model that describes sensors, actuators, and processing resources. It also offers a framework based on that model, to support queries and perform requests to actuators. On the other hand, [46] proposes a metadata framework inspired by EPCglobal network [47] to enable plug and play Wireless Sensor and Actor Networks (WSAN) into the Internet. The metadata managed by this framework allows discovering nodes and provides a list of available interfaces for query/actuating services as well as their application level message formats. There are other many REST-based frameworks [48–50], that facilitate the development of generic RESTful services. Those frameworks are focused on deploying resources on no constrained devices as gateways or servers. This solution is useful for encapsulating functionalities of embedded devices that are not capable of natively running a complete ROA.

## 3. Research Challenges

In the previous Section, the state of the art related to the Model-Driven approaches, semantic models and frameworks to facilitate the development and integration of smart space into WoT paradigm was analyzed. From this study, we have identified some research challenges which should lead to the design of a development methodology for large and heterogeneous smart spaces in the field of WoT in order to be integrated into more extensive concepts as smart cities. We have summarized these challenges in the following points:

- **Reusability and importability (*challenge 1*)**: In the software industry for embedded systems, it is usual to manage many different languages to model components and artifacts that will compose more complex systems. MDE-based development methodologies can provide DSLs to model diverse aspects of embedded systems hiding the plethora of technological platforms in the market as well as increasing their reusability among different deployment scenarios.
- **Modeling constraints and semantics (*challenge 2*)**: The modeling through DSLs have to be restricted by defining a specific syntax that constraints the use of concepts of the language. The models also have to comply with the domain information in which the resulted artifacts will be deployed; thus there is a need of an information model providing context information near to the real-world environment.
- **Role coordination (*challenge 3*)**: In the development of large smart spaces some different roles are involved. To this purpose, coordination mechanisms are a must; a rule of thumb to achieve such coordination is to “speak” the same modeling language, the better if it is an industrial standard.
- **Integration into IoT and WoT (*challenge 4*)**: Currently, IoT and WoT principles are a reference for the construction and integration of networks of embedded devices. The design of Model-Driven development methodologies for smart spaces has to take these architectural pillars into consideration.
- **User-centric programming (*challenge 5*)**: Modeling frameworks can abstract the developer from technical aspects regarding the domain to be modeled. The goal of hiding such aspects is to provide a CASE (Computer Aided Software Engineering) tool focused on increasing the productivity while decreasing the learning effort of a DSL.
- **Assistance to verification procedures (*challenge 6*)**: Related to the previous challenge, the verification procedures allow analyzing the created models to solve inconsistencies both semantically and syntactically. This process has to take place transparently for the modeling tasks without interfering in major development objectives.

We consider that the research challenges described above may be considered through a development methodology that implements solutions for each of them, in order to reach a holistic solution for rapid deployments of smart spaces; such an integrated approach could significantly reduce global resources (financial and human) when developing and deploying large and complex smart spaces. To integrate previous works, we analyzed the existing literature in order to show how the considered challenges have been addressed to date. The result of this analysis has been gathered in a comparative table (see Table 1).

From the comparative analysis, it is noted that related works do not provide a holistic approach we aim at delivering through our proposal. The works of Walter *et al.* [29] and Katasonov *et al.* [16], which are the most complete ones, still have some drawbacks, mainly related to their capability to deploy heterogeneous smart spaces composed of a number of embedded sensorial and actuator devices, as well as their adaptability for generating software artifacts complying IoT and WoT principles. Such drawbacks are tackled in our proposal. Firstly, the design of this approach was based on MDA that provides a set of modeling tools in the environment of a well defined and standard architecture; such feature enables robust mechanisms to create a set of phases and roles managing the whole development cycle of large smart spaces (*challenge 3*).

In order to design a canonical MDA approach, we specified a set of novel DSLs to model different aspects (*challenge 1*). On one hand, these DSLs allow defining the behavior and contextual activities of smart objects from a high level point of view. On the other hand, they enable modeling tools to define functional aspects of business processes from a low level point of view, associated to the previously modeled behaviors.

Additionally, this work extends and improves traditional MDA-based approaches by introducing semantic technologies, which allow solving problems related to traceability as well as verification of consistency and completeness of the models in relation to domain concepts (*challenge 2*). This feature is supported by means of an ontological resource: the Smart Space Ontology (SSO). The specification of SSO was inspired on previous works as OWL-S or SSN. Specifically, SSN was used as upper ontology to define an essential part of SSO; a novel technique has been used in order to merge already defined ontological resource in SSN and new semantic structures in SSO. Essentially, our approach uses SSO with the purpose of describing specific smart space domains and verifies ECM and SOM models according to those semantic descriptions. This approach differs from other ones trying to extend meta-metamodels, metamodels and, even, models with semantic technologies (e.g., [29]) to verify consistency and satisfiability of DSL designs and their instances (models) through constraints defined in ontologies. It is also important to note that both approaches can coexist.

Another improvement provided by ROOD methodology with regard to previous MDE-based approaches ([15,16,28,51]) is its capability to model functionalities of the system in order to generate artifacts that perfectly adapt to REST architectural style (*challenge 4*). This feature facilitates the convergence between embedded pervasive networks previously disconnected among them [43].

Finally, ROOD methodology provides a toolkit based on graphical modeling (e.g., for high level behavior sketching and business processes definition), that makes easier the development and deployment of smart spaces independently of their dimension and device heterogeneity (*challenge 5*). The main processes performed by the ROOD toolkit consist of user guidance during modeling, both at CIM and PIM levels, that ensure well-formed models according to the restrictions and constraints defined by metamodels (*challenge 6*). Besides, it allows verifying the consistency of models according to the domain specification stored in the KB of the smart space. The following Section provides a detailed overview of the ROOD methodology.

## 4. The Resource-Oriented and Ontology-Driven Methodology: Principles and Architecture

ROOD methodology provides the guidelines to develop smart spaces under an Internet-connected resource-oriented approach (following the paradigm of the WoT). In ROOD, a *resource* denotes an entity, which is managed by a smart object, encapsulating simple or complex business processes through a RESTful architectural style. Those business processes are in charge of setting sensing and actuating tasks up, as well as accomplishing the processing and reasoning of the data gathered through the available sensors. The ROOD's design has been conducted by three main design objectives derived from the challenges explained in the Section 3:

- *The creation of a model-driven framework for rapid prototyping of large and complex smart spaces (challenges 5)*: the methodology has to provide the guidelines and tools to model the workflows that characterize the behavior of the smart system from their high-level description, to the subsequent alignment of those models into information and processing ones, closely related to the sensing and actuator platforms currently available at the market (e.g., motes, mobile devices, specific sensors, *etc.*).
- *The improvement of traditional MDE approaches*, *particularly of those focused on MDA*, *by semantically supporting each development phase (challenges 1,2,3 and 6)*: the methodology has to provide a set of models extending from CIM level to PIM and PSM levels, and specific semantic technologies based on ontological resources that allow conceptualizing different aspects of the system to be developed, in order to verify the consistency and integrity of models in each stage, as well as supporting bilateral transformations.
- *The integration of smart spaces based on Internet of Things principles (i.e.*, *implementing mechanisms for interactions among smart objects) into the Web of Things (i.e.*, *using mechanisms to facilitate the development of Internet services and Web resources) (challenge 4)*: the methodology has to be able to automatically generate the artifacts providing RESTful services, that are to be deployed on embedded networked devices or gateways encapsulating specific embedded platforms.

Successfully addressing these objectives implies applying concepts from a number of different technical fields related to software engineering, semantic technologies, and pervasive embedded networks, among others.

The ROOD methodology addresses the development of smart spaces from two different perspectives: (a) the *contextual activities*, which specifies the behavior of the resources (sensor, actuator, and interfaces for human interactions) used within a smart space and the relationships among them, and (b) the *smart object*, which provides a deployment perspective of the system involving information and processing models characterizing sensor and actuator entities within the smart space and its association with RESTful services. These views can be linked to the CIM and PIM levels of a typical MDA-based approach, respectively. The ROOD methodology includes models related to both levels that encompass the mentioned features: (a) The *Environment Context Model* (ECM), and (b) The *Smart Object Model* (SOM). These models are instances of a DSL, the Smart Space Modeling Language (SsML) that was designed as an UML profile. Additionally, the modeling processes concerning those models are enriched through semantic technologies; concepts represented both in ECM and SOM are aligned to semantic contents that are stored in Knowledge Bases (KB) and defined according to an ontology called *Smart Space Ontology* (SSO). In that way, the ROOD methodology takes advantage from ontological resources to verify the completeness and consistence of ECM and SOM models according to the semantic description of the domain system; consequently the verification mechanism optimizes the model-to-model transformation processes from ECM to SOM.

In the following sections, an overview of the principles and architecture of the ROOD methodology is firstly provided by means of an explanation of every part of its architecture as well as the phases involved in it. Firstly, the model-driven foundation of the ROOD methodology is explained, including the UML profile, in which the SsML is based, as well as its instances, ECM and SOM. Secondly, an explanation of how semantic (ontology-based) technologies are used along ROOD methodology is provided. Finally, a complete description of the stages of the ROOD methodology is given.

### 4.1. The Smart Space Modeling Language: An Overview

In this section we give a brief description of the features of the Smart Space Modeling Language (SsML). The SsML is a UML 2 profile whose major objective is to support the two main models of an MDA-based methodology, CIM and PIM that, in ROOD, correspond to the aforementioned ECM and SOM, respectively.

OMG has standardized some UML profiles that deal with real-time embedded devices [52,53] and services-oriented architectures [24]. Those profiles addressed issues related to some aspects of smart spaces as embedded devices (e.g., tasks scheduling or hardware resources allocation) and service-oriented mechanisms (e.g., service producers and consumers, contracts or interfaces). Our approach requires a single solution addressing both service orientation and hardware modeling. Moreover, that solution requires supporting smart object integration in a heterogeneous, dynamic and resource-oriented platform. Keeping in mind this challenge, we have defined SsML, whose major goal is to support resource modeling within Resource-Oriented Architectures (ROA), from the twofold view of (a) activities performed in a smart space to (b) internal resources and tasks implemented into specific devices.

The definition of SsML depends on the MDA architecture that is stratified in four abstraction levels (M0 through M3). M0 contains instances of data for a specific platform; M1 is where the system's models are defined; M2 specifies the DSLs that take part in the definition of models at M1. Finally, M3 defines the Meta-Object Facility (MOF), that establishes the basis for different modeling languages.

**Figure 1.**
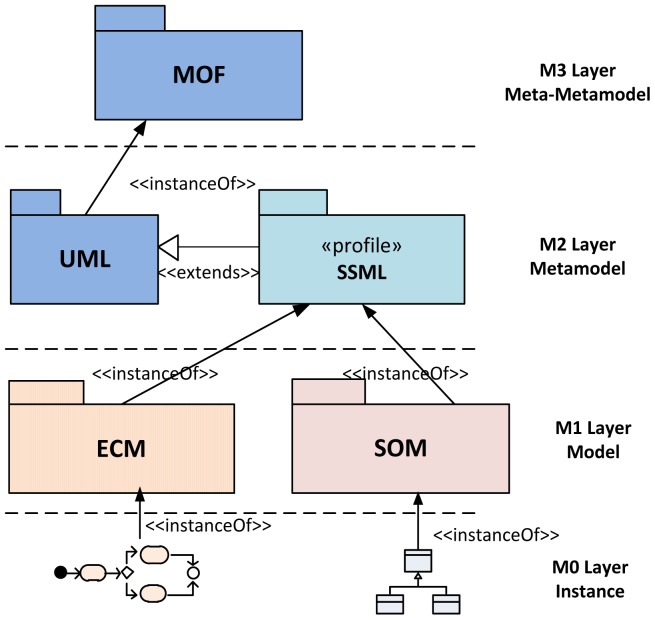
MDA's perspective of SsML.

Figure 1 shows the logical position of the SsML in the MDA methodology. As it can be seen, the SsML is hosted in M2 layer and extends the UML metamodel; SsML uses the extension mechanisms defined in the UML 2 specification in order to create an own profile that defines every necessary element (entities, relations and interfaces) to model the smart space. The SsML is the origin of the model-driven development supporting the ROOD methodology, since both ECM and SOM are instantiated from it. The ECM is an instance of SsML that allows modeling the behavior of a smart space in terms of activities (workflow), relationships between activities and events triggering. The SOM is also an instance of SsML that allows modeling “things” or smart objects taking part in the smart space, including their functionalities and business processes offered by means of RESTful services.

In summary, ECM and SOM provide a set of modeling tools enabling ROOD methodology to build complex smart spaces from two different viewpoints. As said before, the viewpoints defined in both ECM and SOM correspond to the CIM and PIM of the MDA. From those viewpoints, instances of the smart space can be defined through models that are represented in the form of diagrams with a specific notation. In ROOD methodology, the drawing schema of the diagrams is restricted by the constraints specified in specific viewpoints. Moreover, domain concepts represented in those diagrams are validated through semantic technologies supported by ontological resources that determine what can be represented according to the participants belonging to the smart space (e.g., sensor/actuator devices and users) and to the functionalities they can provide or consume (e.g., sensing or actuating resources). Such semantic information will be stored in Knowledge Bases and will depend on an ontological resource, as shown in Section 4.4. In the following sections we describe the ECM and SOM and all the pieces of the SsML metamodel, which establishes some modeling constraints for each one of them.

### 4.2. The Environment Context Model

According to the MDA specification, CIM models are used to represent the environment and the requirements of the system they model, without referencing any detail about the implementation of the internal processes and tasks [23]. The ROOD methodology proposes the Environment Context Model (ECM) in order to address modeling aspect corresponding to the CIM. The ECM models scenarios in which activities, performed in a smart space, are depicted in a logical structure similar to UML 2 activity diagrams. The objective of activity diagrams is the modeling of *the sequence and conditions for coordinating lower-level behaviors* [18]. They are traditionally called control flow and object flow models. The behaviors controlled by these models are scheduled by control structures that take place when actions finish their execution, when objects and data become available or when external-to-the-flow events happen [54].

**Figure 2.**
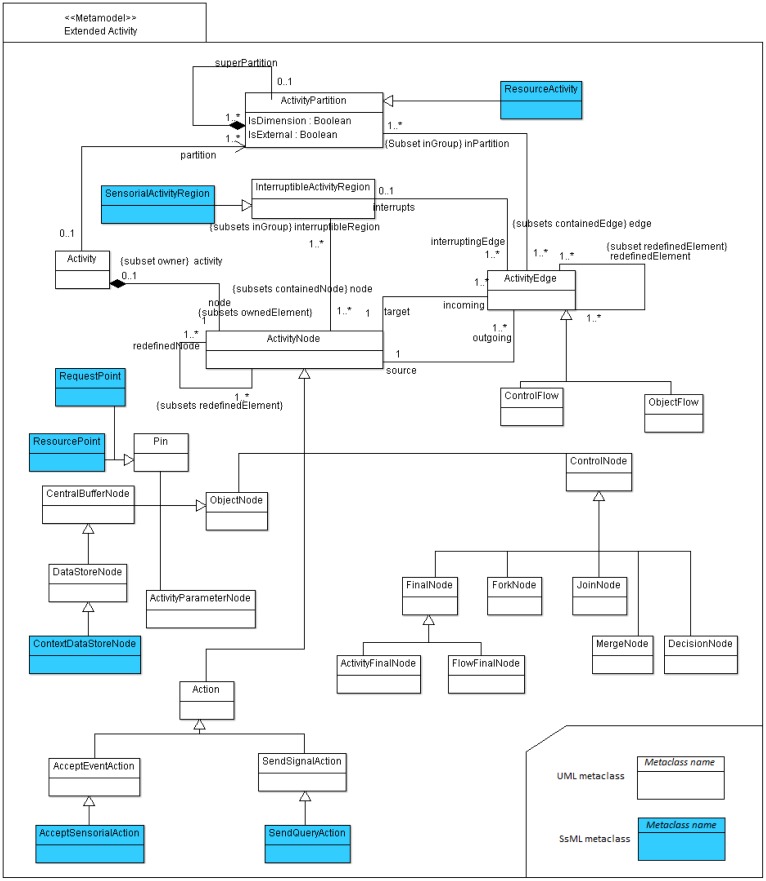
Part of the SsML profile focusing on the extension of behavioral packages of UML 2.

Anyway, the characteristics of UML activity diagrams do not properly tackle the objectives of CIM as established in the MDA specification, *i.e.*, the modeling of interactions between business processes and the interchanged data involved in the environment to be modeled, independently of the platform which will be used to deploy the defined processes. We consider that the activity diagram, as it is defined in UML 2.2 Superstructure [54] does not natively support the definition and graphical representation of participant roles, interfaces, data persistence or type of messages and other behaviors that should characterize a RESTful system managing a smart space. For example, by using UML 2, it is not possible to draw resource providers, resources consumers, request neither types nor regions that are influenced by sensor events as a result for an asynchronous request. With the aim of solving this drawback that restricts the expressiveness of UML 2 for modeling REST-based smart spaces, we propose to create a unified syntax by extending activity concepts of the UML 2 metamodel. For this purpose, SsML extends the behavioral set of packages from the UML superstructure [54], in order to add relevant aspects, which will increase the usability of the activity diagram offered by UML 2 metamodel. This particular extension is shown in Figure 2.

The extension shown in Figure 2 can be considered as a stereotype of the existing metaclasses. The *stereotype* is the most common extension mechanism provided by the UML 2 specification. Stereotypes are used to expand the core elements of UML in order to extend and classify associations, inheritance relationships, classes, and components. In this case, the extension of the activity packages, as it is shown in Figure 2, does not strictly follow the general method to define a UML 2 profile. In the UML 2 superstructure specification [54] a stratification of language units (from L0 to L4) is defined as the foundation for defining compliance in UML. At each layer, elements of the UML metamodel are defined with an incremental complexity through a mechanism called *package merge*. The usual method for defining UML profiles extends language units residing at L2. However, SsML extends language units residing both at L1 and L2 in order to gather all the required elements to define enriched activity models.

A brief description of every stereotype shown in Figure 2 is provided in Tables 2–4. Associations and constraints are applied to the stereotypes of the metamodel in order to indicate restrictions. Constraints can be defined in any language, as natural language or programming language. A formal way to express constraints is by using OCL (Object Constraint Language) [55]. In order to facilitate the understanding to non-expert readers, natural language is used in Tables 2–4 to define constraints for each element extended from UML superstructure. Additionally the graphical notation associated with each stereotype is included. This paper does not provide a detailed semantic description of each node type but only a brief description, sufficient to understand the activity aspects of SsML. As described in [54], we have classified the extensions performed over activity packages in the following groups: *Graphic Nodes*, *Graphic Paths* and *Other Graph Elements*.

**Figure 3.**
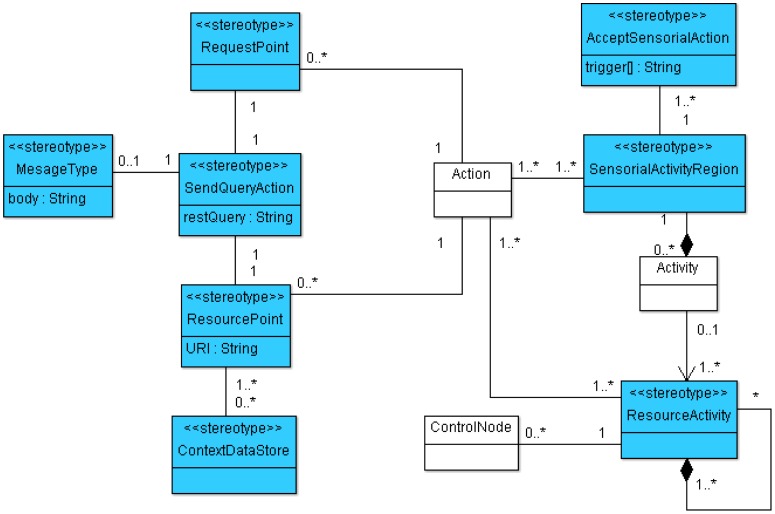
The ECM's viewpoint of the SsML subset extending behavioral concepts from UML2 metamodel.

The enhanced semantic provided by SsML is mapped over a visual modeling language that we have named Environment Context Model (ECM). From the UML extension described previously, SsML establishes the foundations for the definition of the ECM, including its syntax and elements involved in an activity diagram for a smart space based on REST communication mechanisms. ECM's viewpoint of the concepts in SsML has been defined in order to establish the constraints that drive the modeling of ECM diagrams. This viewpoint is shown in Figure 3.

### 4.3. The Smart Object Model

The next stage, according to MDA architecture [23], corresponds to PIM models. The model proposed by the ROOD methodology at this level focuses on defining the system functionalities and providing access to interfaces to use them, without concerning itself with the technological details of the platform on which the modeled artifacts will be deployed. For example, ROOD allows defining complex business processes (e.g., management of physical activity of users in a gym setting), which orchestrates low-level tasks performed by a specific platform (e.g., management of sensors and actuators). Those business processes are scheduled according to the traditional service-oriented paradigm that is mapped over RESTful services. Thus, REST interfaces are actually offered to external entities in order to facilitate the use of the smart object functionalities. This model is called Smart Object Model (SOM) and it was designed for modeling different features of the infrastructure involved in a smart space. SsML provides key pieces that facilitate the design of the semantic and syntax of SOM.

**Figure 4.**
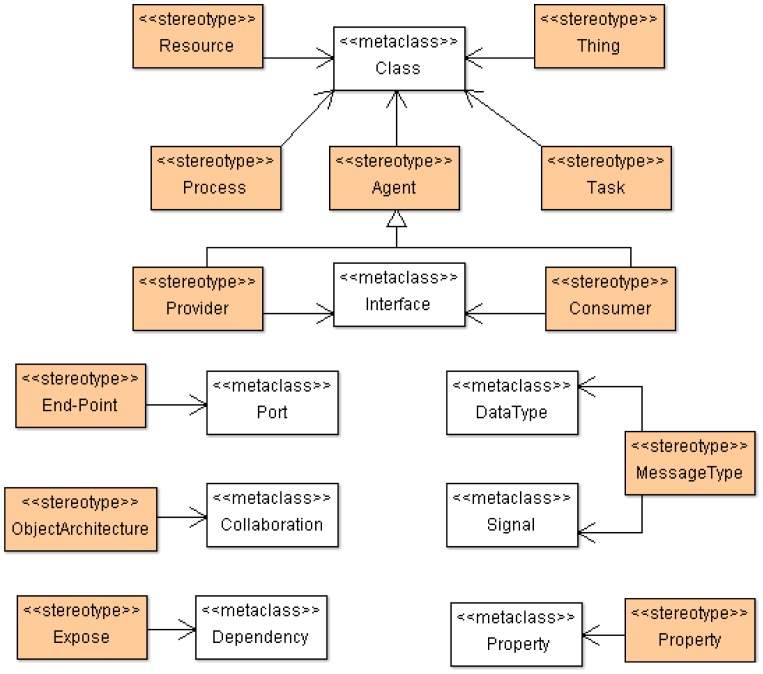
Part of the SsML profile focusing on defining a metamodel for modeling diverse functional aspects of the smart spaces.

SsML uses UML's general profile mechanisms [18] in order to set up the SOM's foundation at M2 layer of the MDA architecture (see Figure 1). This is not a first-class extension mechanism, *i.e.*, it does not modify the existing metamodels. The aim of the SsML profile is to adapt existing metamodels of UML for the particularities of smart space environments. The extension defined by SsML for this purpose is shown in Figure 4.

Stereotypes defining the required concepts for creating SOM are described in Table 5. In this case, SOM will be represented as a class diagram. Thus, instances of specific stereotypes will be draw as stereotyped classes.

**Figure 5.**
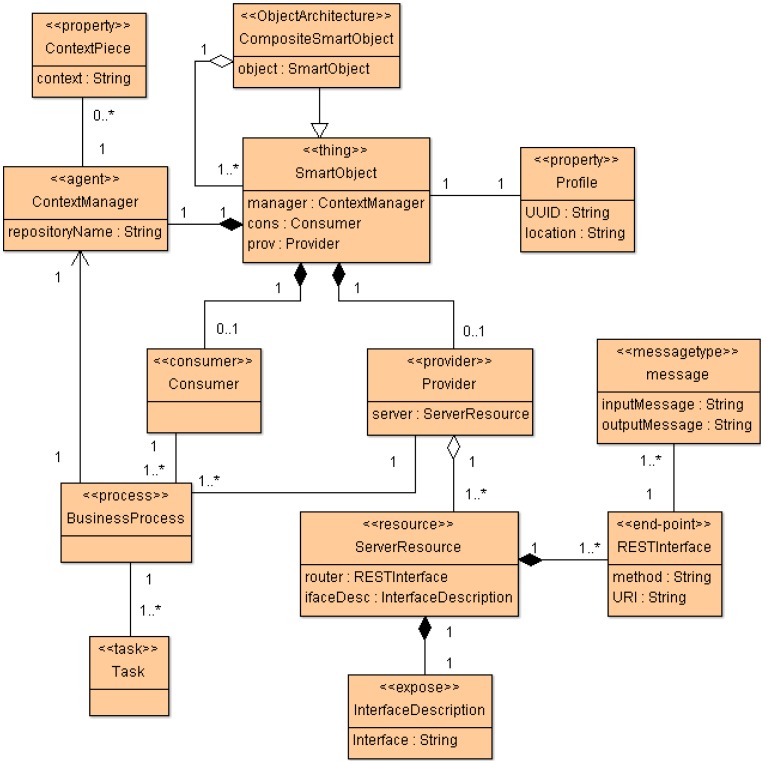
SOM's viewpoint of the SsML metamodel.

In the same way as ECM, SOM is a particularization of the SsML metamodel, *i.e.*, instantiations of the stereotypes are defined in SsML, which in turn defines a specific semantic and syntax. The SOM viewpoint defines several constraints that aim at guiding users to build well-formed models. This viewpoint is represented in Figure 5.

SOM is focused on modeling participants (smart objects) working collaboratively in smart spaces to reach a common objective. The major features of SOM for modeling resources, processes and interacting smart objects are the following:

- SOM is able to model communities of interacting smart objects (*Things*), specifying the roles played by both participants (*Provider* and *Consumer*) and resource interfaces (*End-point*).
- *Resources* are provided by smart objects (*Things*), which are in charge of implementing underlying services characterizing the resource behavior and its state. Smart objects can host more than one resource.
- SOM is especially designed to be supported by ROA-based approaches. This characteristic facilitates the generation and deployment of artifacts for RESTful architectures.
- Resource interfaces are specified through *End-points*, which are defined through RESTful interfaces, characterized by an URI, a HTTP method (GET, PUT, POST and DELETE) and message types for inputs and outputs.

### 4.4. Semantic Technologies for Integrity Verification of Deployment Scenarios

As explained above, ECM and SOM offer a set of modeling tools for intelligently describing the relationship between different real-world entities available in the Smart Space domain (e.g., sensors, actuators, services, resources, physical spaces, *etc.*), where each particular instantiation of these entities can be considered as a *deployment scenario*. ROOD methodology proposes a semantic-based approach for describing these scenarios, achieving not only a high degree of integrity in their definition but also in the definition of the ECM and SOM models exploiting them. This integrity assurance is mainly supported by the *Smart Space Ontology* (SSO), which is structured as three sub-ontologies modeling different sets of Smart Spaces entities:

- The Domain sub-ontology describes the physical characteristics of a Smart Space, populated with Smart Objects managing devices with sensing and actuating capabilities; it is mainly based on an extension of the W3C's SSN (Semantic Sensor Network) ontology [21].
- A Service sub-ontology that defines the necessary entities and properties for modeling a service oriented architecture. For example, it defines from composite processes to simple tasks, as well as elements to orchestrate them.
- Finally, the Resource sub-ontology allows encapsulating service functionalities through REST interfaces, *i.e.*, it defines every entity necessary to map smart objects services into RESTful style resources.

NeOn Framework [56], a novel methodology for ontology design and development, has been used for building SSO. This framework proposes a set of mechanisms for collaborative ontology development, reuse of ontological and non-ontological resources, as well as the evolution and maintenance of networked ontologies. NeOn does not offer strict rules but a set of suggestions about different scenarios covering the most common situations, e.g., when existing ontologies have to be modified through a reengineering process, or the process of alignment, modularization, or integration with non-ontological resources.

The design of SSO was basically conducted by two mechanisms described in NeOn methodology:

- Reusing Domain Ontologies: this mechanism is focused on integrating *general* or *common* ontologies into a *host* ontology, in order to address the modeling of specific entities. The first activity of this mechanism consists of choosing the common ontology (or ontologies) that best fits the characteristics of the problem to be solved. The second activity consists of customizing the selected common ontology (or ontologies) according to the domain and to integrate it into the host ontology.
- Ontology Modularization: this task consists of identifying those parts of an ontology that can be considered as independent modules (*i.e.*, sub-ontologies), while they are interconnected to each other. Ontology Modularization facilitates the reuse and maintenance of ontological resources.

**Figure 6.**
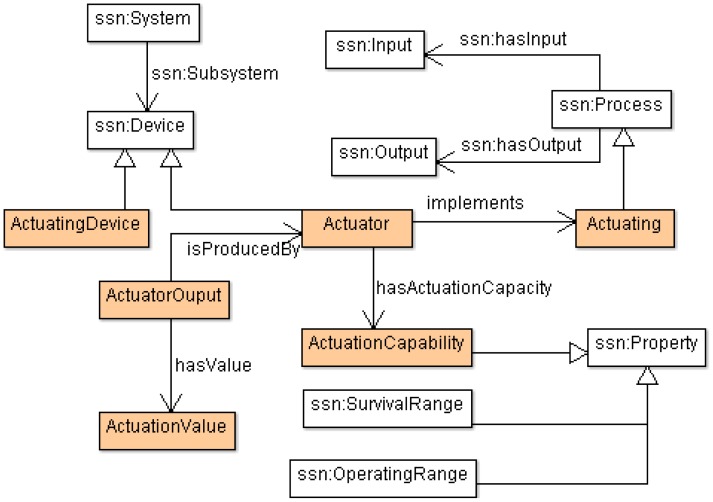
SSN extension introducing concepts related to the actuator conceptualization.

Taking into account these mechanisms, SSN ontology was selected as a ground model for building the *Domain* sub-ontology. SSN models some of the most important domain concepts of a smart space (that is, sensor and actuator devices as well as their capabilities or their integration in a specific deployment). From an exhaustive analysis of the SSN ontology, some drawbacks were found: although SSN provides enriched semantics to characterize sensors, it has no semantics to define actuators and all concepts around them, being essential elements for conceptualizing high interactive smart spaces (*i.e.*, processes, capabilities and output actions). Reengineering methods specified by NeOn were used in order to evolve the SSN ontology by adding those new concepts. That evolution consisted of adding classes and relations for defining actuators belonging to a smart space; the extension that conducts such evolution is shown in Figure 6.

NeOn Methodology was also used to specify the *Service* and *Resource* sub-ontologies of the SSO from scratch and to align each other with the *Domain* sub-ontology. This way, we enabled the SSO to capture and specify environment requirements and to verify the ontology with respect to the requirements it has to fulfill. Those requirements are gathered through a conceptualization analysis of the smart space that is described in Section 6.2.

**Figure 7.**
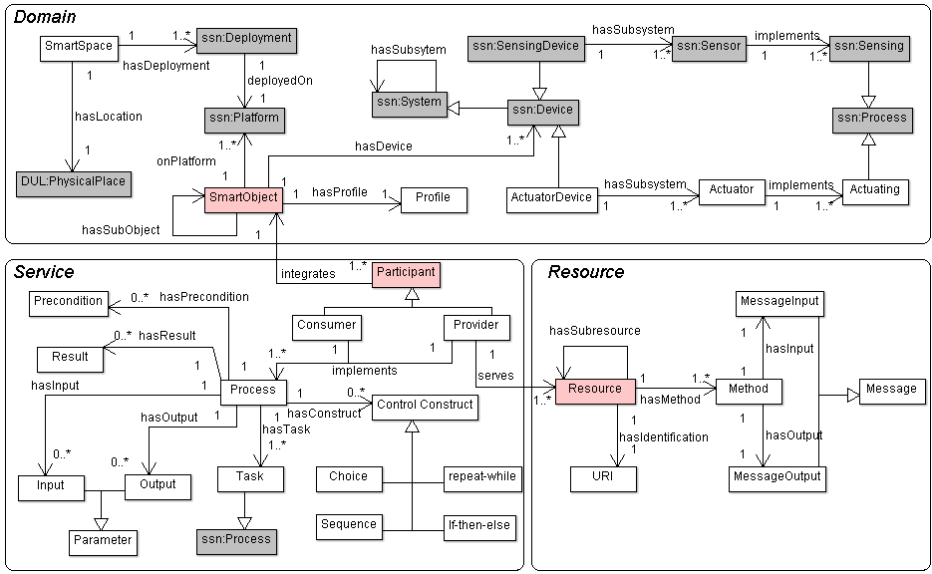
Overview of the Smart Space Ontology: modules, classes and relations. In grey color, classes coming from SSN ontology.

The major entities included in each sub-ontology of the final version of SSO are presented in Figure 7, where grey boxes represent SSN entities. *hasDeployment* and *hasLocation* properties are used to relate SSO entity *SmartSpace* and SSN ontology. Apart from the extension depicted in Figure 6, SSO smart objects links to SSN taking into account that a *SmartObject* is built on a specific *ssn:Platform* (*onPlatform* property) and it is composed of several kinds of *ssn:Device*s (*hasDevice* property). The Service sub-ontology is aligned with the Domain one by means of *Participant* entity (*integrates* property), which relates *SmartObject* (and their *ssn:Sensor*s and *Actuator*s) to business *Process* managing low level *Task*s. *Resource* is the main class in Resource sub-ontology; this class is directly linked to sensing and acting processes offered by *Provider* agents in the Service sub-ontology.

The abovementioned semantic models have been developed as OWL-based documents; each particular deployment scenario to be used at ECM or SOM level would need to be built as RDF triples based on these documents, conforming a specific deployment scenario Knowledge Base (KB).

**Figure 8.**
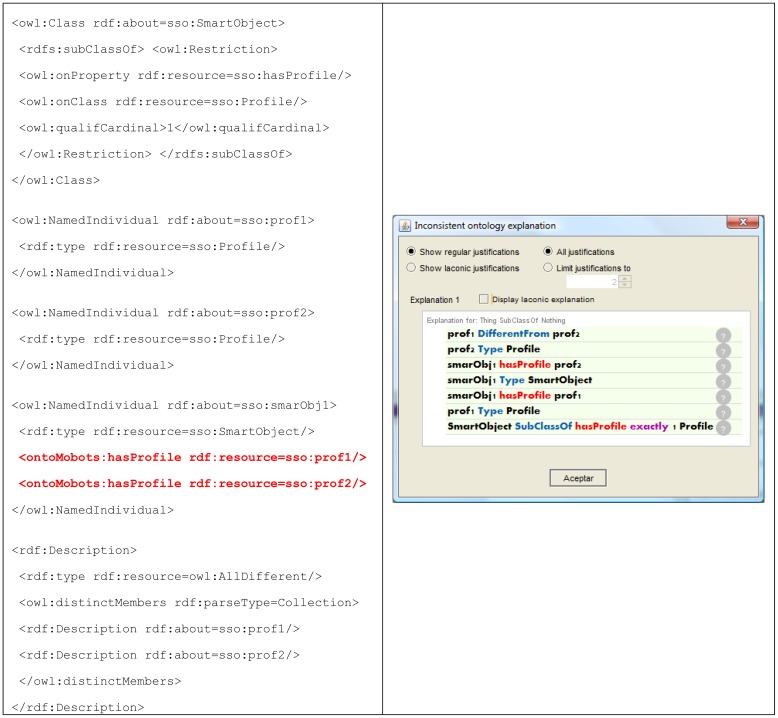
Example of inconsistent deployment scenario KB (consistency checking).

This semantic modeling of the deployment scenario for smart spaces brings several useful advantages for the MDA methodology (both at CIM and PIM level). Using a standardized language (OWL) and integrating existing and well-known models improve the compatibility of ROOD methodology with other semantic tools. It also guarantees consistency of the deployment scenario entities used in ECM and SOM, as general purpose reasoners can be invoked in order to support semantic engineers during KB filing (satisfiability checking according SSO schema), and business and software engineers during model creation (consistency checking according KB contents). In this sense, Figure 8 (left) shows an example where a semantic engineer involved in ROOD methodology tries to assign two different profiles (*sso:prof1* and *sso:prof2*) to the same smart object (*sso:smartObj1*) but a cardinality restriction set in SSO states that each smart object must have just one profile (<*owl:qualifiedCardinality*>*1*<*/owl:qualifiedCardinality*>). Besides detecting these inconsistencies, recent researches address *explanations generation* that can be used as a guide for ECM and SOM models designers [57]. Figure 8 (right) shows the explanation to the previously presented inconsistency (explanation offered by Protégé 4.2).

Regarding this ontology design process, future works may consider also integrating other well-known models as, e.g., OWL-S [40] for describing semantic web services in general (or RESTfulGrounding ontology [58] for modeling a RESTful architectural style).

## 5. Phases of the ROOD Methodology: From Models to Code

This section presents the different stages of the ROOD methodology involving the elements of the architecture defined in Section 4. This guideline highlights the traceability between the concepts presented in ECM and SOM metamodels, as well as the mechanisms to verify models delivered in each stage.

As discussed in the previous section, any development methodology based on MDA consists of three main phases: i) Computation Independent Model (CIM); ii) Platform Independent Model (PIM); iii) Platform Specific Model (PSM). Along these phases, MDA manages to separate the conceptual design (focusing on functional requirements of the system) from the platform features (defining no functional and technological aspects of the underlying architecture).

Generally, MDA approaches specify transformation rules between PIM and CIM but just traceability relations between the requirements on CIM models and the concepts of PIM and PSM models. Very often, the own nature of CIM models prevents the creation of direct transformations to PIM models since, while CIM describes functional viewpoints (*i.e.*, behavioral aspects of the system environment), PIM and PSM defines architectural and deployment aspects in order to accomplish the requirements of the system. The ROOD methodology proposes to solve this alignment issue by means of a chain of transformations between ECM and SOM models, belonging to CIM and PIM, respectively.

**Figure 9.**
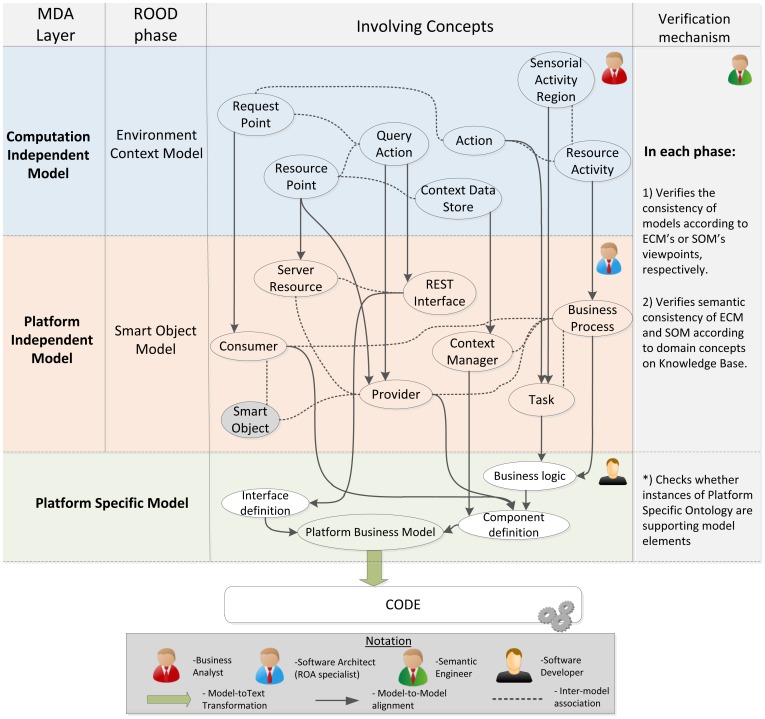
ROOD methodology phases: traceability, participant roles and verification mechanisms.

Firstly, we identified a set of traceability relations between concepts in both models taking into consideration the elements defined in their viewpoints (see Sections 4.2 and 4.3). In Figure 9, the major concepts that have to be modeled in each ROOD's stage as well as the traceability between ECM and SOM elements are represented. Additionally, the processes for model verification are briefly described.

The concepts shown in Figure 9, which characterizes the ECM and SOM of a smart space, are defined on viewpoints described in Sections 4.2 and 4.3, that derive from the UML profile specified by the SsML metamodel. Such viewpoints set restrictions of use for different entities in each stage of the ROOD methodology, as well as their relationships.

Moreover, semantic technologies play an important role within the ROOD methodology, which take all its potential from the SSO. The SSO was essentially designed in order to adjust the modeling process to specific smart spaces through Knowledge Bases, gathering concrete domain information about different scenarios. The entities specified in SSO are projected on concepts of ECM and SOM models. Those relationships are indicated in Table 6, which shows relevant concepts of the SSO and how they are related to entities defined in ECM and SOM viewpoints. The definition of those relationships facilitates the verification of integrity and completeness of models in relation to the domain information stored in Knowledge Bases.

Traceability relations defined in Figure 9 and Table 6 allow for generating specific elements from one model to another (model-to-model transformation).Thus, for example, concepts such *Request points* in ECM will be transformed into *Consumers* in SOM, or *Context Data Store* in ECM will be transformed into *Context Manager* in SOM. The transformation process can be performed totally or partially, that is, whether the transformation among entities needs total or partial human intervention

The ROOD methodology provides a well-specified set of modeling tools and common terminology promoting collaborative work among individuals of heterogeneous development teams without compromising global project resources and efforts. It is important to highlight that the ROOD methodology is flexible and its stages are loosely couple. This characteristic facilitates the involvement of a variety of kinds of professionals collaborating and working jointly in the development chain, e.g., (i) business analyst; (ii) software architect, specialized in ROA; (iii) semantic engineer; (iv) software developer.

Before beginning with the methodology, it is important to identify each element of the smart space by analyzing the physical places that will be populated by smart objects. Meanwhile, every smart object in such smart space is supported by one or more platforms with a set of sensor and actuator devices; these sensor and actuator devices are responsible for leading the expected behavior to the smart space. Such behavior is conditioned to the business processes and underlying tasks (e.g., those managing sensor and actuator work) that are performed by the devices associated with the smart space. In ROOD, these business processes are characterized as services managed by *agents* playing two different roles: *consumer* or *providers*. Finally, services exposed by smart objects to be consumed by other entities belonging to the smart space are encapsulated into *resources*, which are defined according to the REST architectural style. The ROOD methodology follows the most accepted REST implementation in which, for each resource, it is assigned a Unique Resource Identifier (URI), one or more HTTP method (GET, PUT, POST or DELETE) and specific formats for input and output messages (usually XML, RDF or JSON). The analysis of the domain model described before is to be carried out by semantic engineers supported by business analysts and software architects who collaborate in the definition of the technical parameters to be subsequently collected in the smart space's Knowledge Base.

Once the Knowledge Base of the smart space is instantiated, the modeling phase of the ROOD methodology can start. Firstly, business analysts have to model the smart space using the elements defined in the ECM's metamodel. In this stage, only behavioral information of the smart space is represented without concerning the underlying platform, e.g., activity threads, actions, transitions between actions, and smart objects involved in that activity context.

The information contained in ECM models is used to partially generate the models in the next stage, SOM, whose construction is conducted taking into account the viewpoint described in Section 4.3. This stage should be managed by software architects specialized in resource-oriented architectures, who will take advantage of the information from the ECM to model agents (providers or consumers of services), business processes and tasks. Moreover, it will be needed to set up the information system to manage context information that will have an influence in the current and future behavior of the smart objects. Finally, the involved software architects will design the necessary architectural elements that will offer the smart object resources as RESTful services. This step will provide the key piece to integrate the smart space into a WoT paradigm.

The models created in previous steps will be subjected to a verification process to check the consistency and integrity of the entities, relationships and other semantic information represented in them according to the domain information stored in a scenario Knowledge Base.

The final step consists of generating program code from SOM models. For this aim, the elements represented in SOM models are filtered through a model-to-text transformation mechanism. The percentage of generated code can vary depending on the platform but in any case, it will be totally generated. Therefore, a software developer is required to complete existing gaps in the code (e.g., configuration parameters for hardware peripherals or specific information to integrate devices into a communication infrastructure).

It is important to highlight that the transformation specified in the ROOD methodology (ECM-to-SOM and SOM-to-PSM/code) is conducted by mapping rules that in some cases are almost automatically generated and only partially automated in the remainder cases.

## 6. Validation Case Study

ROOD has been partially developed to support research in a large Spanish cooperative project named Technologies for the Hotel of the Future (THOFU Project) [59]. Among its various objectives, THOFU aims at delivering technology to facilitate the deployment of context-aware services in smart hotels to provide an enhanced visitor/guest experience. Within the project, several prototyping scenarios are being designed; these scenarios are conceived to scale into real-world deployments. We following present one of these scenarios, which will be subsequently used to demonstrate how ROOD is applied to facilitate the set-up of a specific smart space technological layer and the services built on it.

*Motivation Scenario*: the smart space used to demonstrate the feasibility of ROOD methodology is a smart hotel. This smart space may be abstracted as a composition of private and common spaces populated with a physical layer of structural, stationary and mobile smart objects, which connect to a virtual augmentation service layer handling services from different providers. People with different service needs and privacy spheres will be users of the smart hotel. With respect to its structure, the hotel has a lot of differentiated areas: rooms, restaurants, welcome zones, common areas such as corridors, stairs or elevators, parking spaces, outdoor spaces, sport facilities, operational zones (kitchens, offices, *etc.*), convertible meeting rooms, *etc.* In all of them, pervasive technology may facilitate the delivery of adaptive services and personalized attention to the visitor, and may help to monetize and optimally manage the available resources.

For this case of use, we have chosen to prototype a *smart indoor gym* by means of the modeling tools provided by the ROOD methodology. Our smart indoor gym will be ready to support a wide range of behaviors and services: (a) visitor seamless identification; (b) personalization of the training session; (c) user's performance monitoring, for posterior personal evaluation and provision of health/nutrition tips; (d) immediate alert to medical services if an unexpected emergency situation occurs; (e) persuasive entertainment throughout the training session; (f) marketing meaningful resources through non-invasive interfaces (e.g., health-related products or sport events) or (g) control of the ambient parameters of the space for optimal service and sustainability.

As a whole, the smart indoor gym makes a complex networked infrastructure in which a number of smart objects are involved, consuming and providing many resources related to the user and environment's contexts. In order to simplify this case study, we will focus on a single service to be provided in the smart indoor gym: the *medical alert service*, which monitors the user's health status and detects and reacts to potential emergency situations.

The following section describes the development process of this service in the smart gym, through the stages proposed by ROOD methodology to model the entities managing the behavior and resources of the involved smart objects.

### 6.1. General Description of the Alert Medical Service

The alert medical service is a basic service, which has to be provided by the *smart gym* to guarantee the healthy practice of physical activity. This service stays completely hidden to the users, since it works in background without needing human intervention. This service is associated with vital signs or activity data from the user, which are analyzed through a smart object called *personal trainer*. The global mission of the personal trainer is to sketch a safe and adaptive work out plan for the user. On the one hand, this smart object can connect and collect data from different biometric sensors, gathering information such as heart rate, body temperature, body humidity and activity. On the other hand, the personal trainer manages a user profile with information about the user's physical condition and health history. These data can be merged with real time ones for different purposes. Additionally, the personal trainer can interact with those exercise machines in the smart gym.

Let us now focus on the emergency detection functionality supplied by the personal trainer. In our validation scenario, when the user enters the smart gym, the personal trainer is downloaded and installed in his/her smartphone; once it is started, it asks for permission to share the user's health profile with the smart gym service. Then, the user is equipped with a *smart T-shirt* (with a biometric belt embedded in it). The smart gym offers to the user the possibility of exercising on a *smart treadmill*. As previously stated, the personal trainer is capable of managing this smart training machine, being able to configure its performance according to the current health status of the user. When exercising, if the personal trainer discerns that the user suffers a sudden change in the vital parameters retrieved from the T-shirt (e.g., unexpected increase of heart rate for the level of exercise) or that an abnormal situation, detectable through the inertial sensors in the T-shirt occurs—e.g., a fall, it generates an alert. In this circumstance, the treadmill is managed to slow down to stop and simultaneously, the notification is forwarded to the medical staff of the smart hotel through an *emergency manager* infrastructure component, which has its counterpart in the medical staff's smartphone.

**Figure 10.**
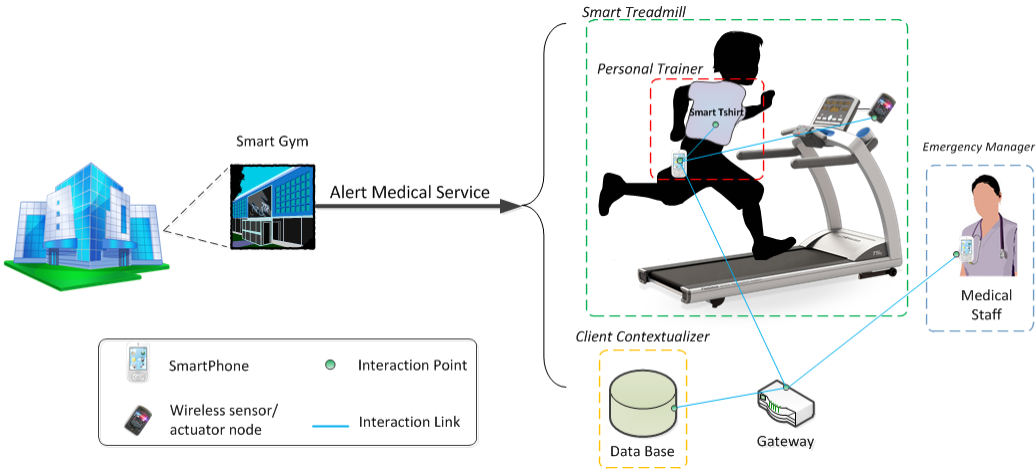
Diagram of the medical service provided by the smart gym. The smart gym is part of a smart hotel.

In short, the components involved in the scenario are the following (Figure 10): (i) a *personal trainer*, composed by the data processing logic to determine if an emergency has occurred from bio and movement signals; it also has activity inference capabilities, to match the health status to the user's movement and detect falls [60]; (ii) a *smart T-shirt*, equipped with the following sensors: heart rate, body temperature and posture/movement inertial sensors; it just hosts the signal processing logic to extract the main features from the signals, which are fed into the personal trainer logic; (iii) a *smart treadmill*, equipped with an embedded platform that performs basic management tasks on the treadmill mechanisms and shown messages; it is managed directly through the personal trainer and communicates its state to the infrastructure; (iv) infrastructure services: a *client contextualizer*, which manages a data base to store user information, in particular about health profiles and health reports for this specific scenario; *emergency manager*, which forwards the alert information to the medical staff's smartphones; (v) a gateway, as a component of the *Acquisition Platform* (The *Acquisition Platform* proposed to set up this case study is also part of the THOFU Project, mentioned previously.), which manages the scenario sensing heterogeneity. The gateway provides a middleware layer for orchestrating the smart objects belonging to the smart hotel, independently of their communication protocol. Components deployed on the *Acquisition Platform* have to provide their functionalities as resources according to a RESTful architectural style in order to preserve the interoperability among elements of the smart space developed by means of ROOD methodology.

Once we have a concise description of the smart space for our case study, let us continue with the practical demonstration of the ROOD methodology in order to prove its feasibility. The following sections describe the modeling process, from ECM to SOM and program code, to develop the alert medical service according to the features specified before.

### 6.2. Conceptualizing the Alert Medical Service

Semantic technologies are a cornerstone in ROOD methodology. As said in Section 5, previously to start the modeling phases, it is essential to carry out a conceptualization analysis of the smart space; this initial analysis allows to achieve good semantic information of the smart space according to the *Smart Space Ontology* (SSO). To this aim, we need to carry out a detailed semantic analysis of the smart space in order to generate an enriched Knowledge Base (KB) of the smart scenario. A well-defined KB will allow fixing the development methodology to a specific scenario and, consequently, to improve the verification and transformation mechanisms during modeling stages. We have designed some guidelines in order to achieve an optimal KB for collecting accurate semantic information; it is not a mandatory to follow these guidelines but they can facilitate the semantic analysis of the smart space. These guidelines, adapted to concepts defined in each module of the SSO (Section 4.4), consist of the following three major procedures:

1. *Analysis of the domain aspects*: This phase encompasses the analysis of those elements building the infrastructure, enabling the smart space to manage the context information necessary for it to deliver its services. In this phase, we need to define the deployment scenario and the physical components in it (platforms, devices, sensors and actuators), and the low-level sensing and actuating processes to be performed.
2. *Analysis of the service aspects*: This phase encompasses the identification of logical components, which adopt two different roles (consumers and producers), as well as the major business processes that they execute. In this phase, we need to identify concepts as the agents involved in expected behaviors of the smart space, and the processes that manage the context and its associated knowledge within the smart space.
3. *Analysis of the resource aspects*: This phase encompasses the identification of resources associated to the processes executed by agents playing a “provider” role, which have to be exposed to external entities in order to be accessed according to a REST architectural style. Thus, it is necessary to formalize those resources taking into account three elements: i) Unique Resource Identifier (URI), ii) one or more HTTP methods (GET, PUT, POST or DELETE), and iii) specific formats for input and output messages.

Following the aforementioned guidelines, Tables 7–9 contains information from a semantic analysis that will be used to populate the KB related to the alert medical service.

### 6.3. Modeling the ECM

As described in previous sections, this stage is intended to model the behavior of the smart space. In order to create models in this stage, we have to use the elements defined in the metamodel specified by the Smart Space Modeling Language (SsML) that are associated with those behavioral aspects of smart spaces (see Figure 2). Moreover, those elements need to be placed in the model according ECM viewpoint (see Figure 3).

**Figure 11.**
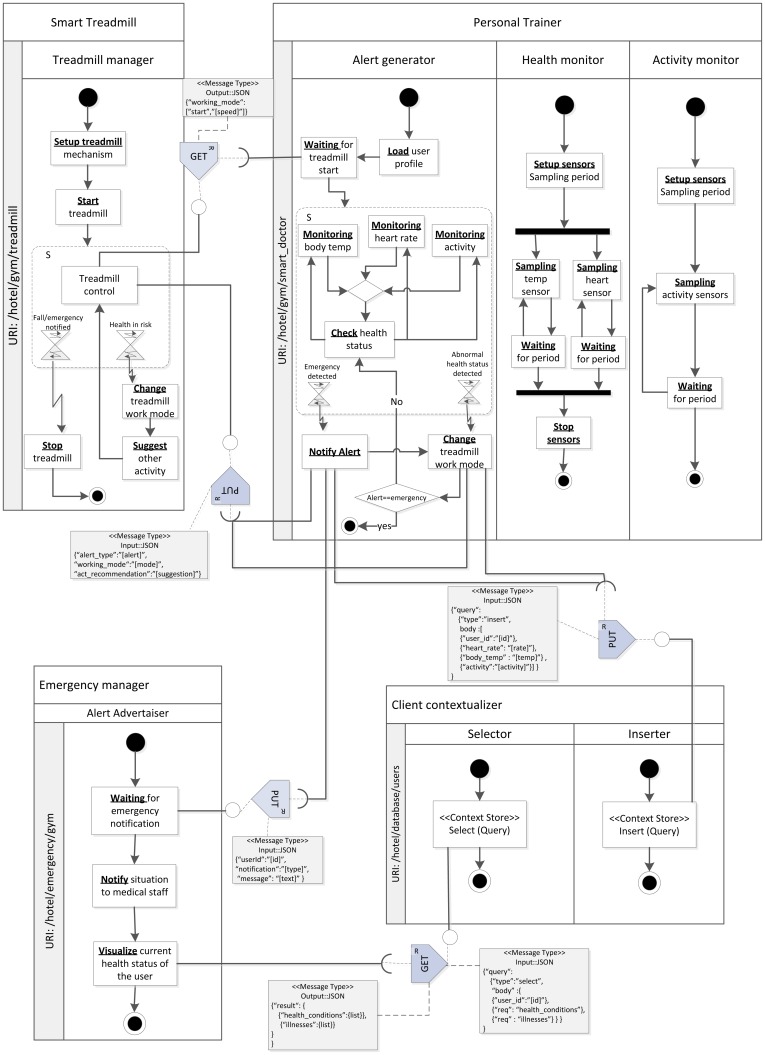
ECM model for the alert medical service offered by the smart gym.

The ECM proposed for this case study is shown in the Figure 11. The graphical notation used in this model is defined in Section 4.2 (Table 2). This ECM shows each one of the smart objects that were identified to take place in the alert medical service (Tables 7 and 8).

Two smart objects, the smart treadmill and the personal trainer, actively interact each other and the latter interacts with the client contextualizer and the emergency manager as well. The last two entities depend on the designed behavior of the smart treadmill and the smart trainer from which they will get context information leading a specific response on both, *i.e.*, storing and retrieving of health reports and notification of alert messages.

Actions characterizing the behavior of entities in the ECM are defined within activity partition (*ResourceActivity* in the SsML metamodel). Those activity partitions represent execution threads that can be performed in parallel. Additionally, activity partition can contain *event sensitive* regions (*SensorialActivityRegion* in the SsML metamodel) which focus on performing actions until the occurrence of a specific event. For example, the personal trainer defines three activity partitions for managing biometric and activity sensors, and a health status analyzer. The last defines a *Sensorial Activity Region* in order to gather and analyze vital and activity signs of the users. The condition to leave this region is to detect an abnormal value in the health report (e.g., exceeded body temperature/heart rate or detected fall) that generates an alert that has to be notified to the treadmill and emergency manager, if the alert is related to an emergency; otherwise, the health report is stored in a data base of users and a health status analysis will be carried out again after passing a period of time when new values of vital signs will be measured. In case of detecting a critical situation for user health, the emergency manager shows a message to the medical staff.

This ECM also models interaction aspects between different smart objects and other entities of the smart space. As said in previous sections, in ROOD methodology, interactions are modeled according to a REST architectural style. Thus, situations in which actions cause a behavior on other entities need to be modeled following the general rules that characterize resources in a REST architecture. For this purpose, SsML metamodel provided elements to model RESTful *interaction points*, that is, *Request Points*, *Resource Point*, *Send Query Action* and *Message Type*. In our case study, there are five different interaction points. For example, it is established an interaction point between the smart treadmill and the personal trainer in order to manage the treadmill mechanism when an alert is detected (to slow down to stop), related to the user's health (e.g., emergency or physical exhaustion of the user). Using ECM, this communication process is modeled as follows: (1) the smart treadmill offers a resource by means of a *Resource Point* that links to a *Request Point* coming from the personal trainer; (2) A REST-based query sent from the personal trainer to the smart treadmill is defined through the element *Send Query Action* that define a method and a URI of the specific resource; (3) An input or output *Message Type* is included to define the specific format and string to be attached to the query.

As shown in Figure 11, the proposed ECM model is adjusted to the information contained in Tables 7 and 8, that is, the ECM model is according to the KB contents which were generated from analysis report of the smart space. Otherwise, if the ECM model is not consistent according to KB contents, the consistency verification would generate an inconsistency diagnoses. Specific mechanisms to carry out model consistency verification are presented in Section 6.6.

**Figure 12.**
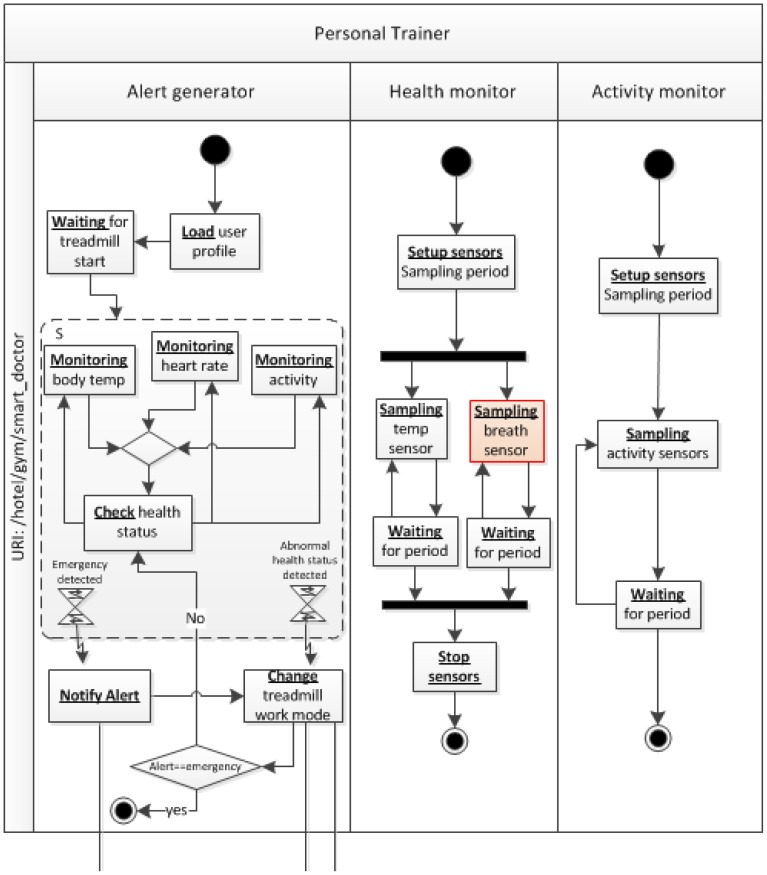
Inconsistent ECM model.

Figure 12 shows an example of an inconsistent ECM model. In this case, the business engineer has included an action within Health monitor thread of the Personal Trainer that tries to sample a breath sensor. However, no breath sensor was inserted into the KB related to Personal Trainer smart object.

On the other hand, ECM model has to be defined according to the viewpoint provided by ECM, which is based on the SsML metamodel. This also guarantees a consistent mapping between the elements represented in this ECM model and the concepts defined in the SOM viewpoint. Consequently, the transformation process to achieve a subsequent SOM model will make better use of the information contained in the previous ECM.

### 6.4. Modeling the SOM

Following the ROOD methodology, the next stage corresponds to the modeling of SOM. As said in previous sections, models generated in this stage are enriched by means of the information defined in models of the previous stage. This is achieved through a model-to-model transformation process whose efficiency depends on the factors commented on Section 5. Thus, in a standard scenario, some artifacts would be available before starting with the modeling of SOM. This facilitates to software architects the design of the SOM models since they will not have to tackle this stage from scratch. The number of artifacts automatically generated will depend on the quality of the KB contents and the design of the previous ECM model. Moreover, whatever the number of artifacts automatically generated is, those will be aligned with ECM entities according to the traceability matrix specified in Table 6.

**Figure 13.**
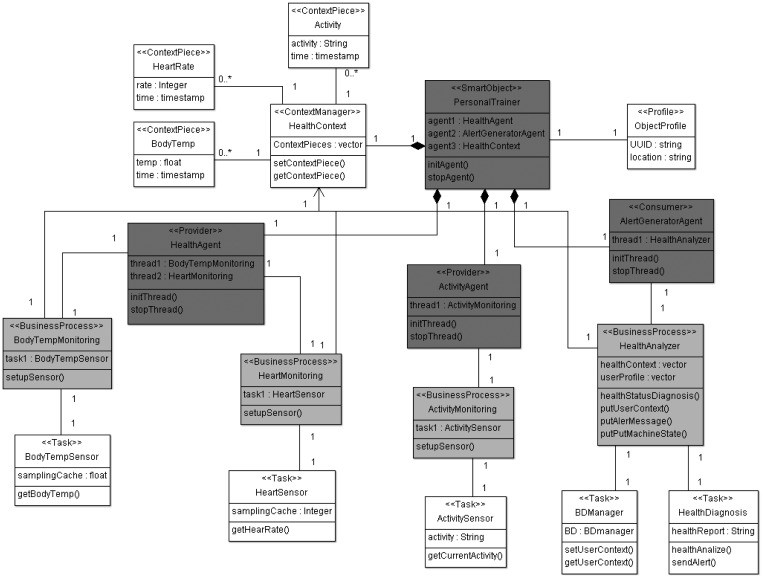
SOM model of the personal trainer belonging to the alert medical service.

**Figure 14.**
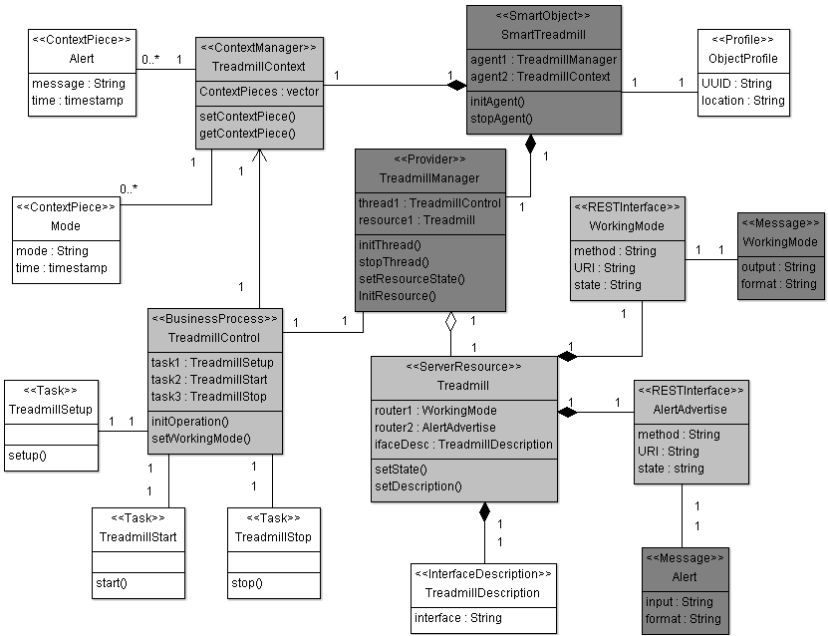
SOM model of the smart treadmill belonging to the alert medical service.

In order to simplify the explanations of the models designed in this stage, let us consider two smart objects: the personal trainer and the smart treadmill. The final SOM models are shown in Figures 13 and 14.

In previous SOM models, the classes in grey color are (totally or partially) automated from the previous ECM model; those classes partially generated are colored in light grey. In that case, the software architect has to complete the semantic information according to technical parameters of the smart space, e.g., identification details for defining URIs completely (*RESTInterface* classes) or context pieces definition to manage context information (*Contex*™*anager* classes). The rest of classes are modeled from scratch and integrated in the model according the SOM viewpoint.

One of the major points in this stage consists of formalizing behaviors modeled in the ECM according to the SOM viewpoint. Thus, actions and activities in ECM (*Action*, *SensorialActivityRegion* and *ResourceActivity* according to ECM viewpoint) are concretized in business processes and task (*BusinessProccess* and *Task* according SOM viewpoint). Each one of the *ResourceActivity* in the previous ECM (*Treadmill* for smart treadmill; *Health Analyzer*, *Activity Monitoring*, *Body Temp. Monitoring* and *Heart Monitoring* for personal trainer) are mapped on *BusinessProcess* classes in SOM. Moreover, some *Actions* and *SensorialActivityRegions* in ECM can be mapped over *Task* classes, or even in *BusinessProcess* classes, in SOM. For example, the *Action* TreadmillControl in ECM model is transformed into a *BusinessProccess* class with the same name and semantics in the subsequent SOM.

Most smart objects populating smart spaces needs to manage information related to their own context and the context of other smart objects and environment. This aspect is considered in the SOM metamodel by means of the *Contex*™*anager* class and the *ContextPiece* associated with it. The *Contex*™*anager* is used by one or more *BussinessProccesses* that get or set context information in the form of *ContextPiece*. To illustrate this characteristic of SOM models, let us look at the model representing the personal trainer. One *Contex*™*anager* (HealthContex™anager) has been modeled for this smart object in order to manage the context information required to perform the processes of personal trainer correctly, *i.e.*, body temperature and heart rate measured in specific instants. There are two activities that are able to provide context information (*Health Monitoring and Activity Monitoring*); this information is then obtained by a third activity of the smart trainer (*Alert Generator*), that processes such information in order to make quick diagnosis about the health status of the user.

An important feature, to be modeled by means of SOM models, is the architectural aspect related to the REST paradigm. This is a keystone to enable a smart object for integration into the Web of Things. To this end, the SOM viewpoint provides the *ServerResource* class, which is associated to one or more interface definitions (*RESTInterface* class) and a description of that interface (*InterfaceDescription*). A *RESTInterface* class defines basic parameters characterizing a RESTful access point, *i.e.*, an URI, a HTTP method, and message types of the interchanged information. The resources modeled in SOM are managed by agents (*Provider* classes), which would play a *server* role in a typical client-server architecture. On the other hand, agents that are not enabled to provide resources but able to access resources to modify its state or receive information from it, are modeled through *Consumer* classes. Those agents would play a client role in client-server architectures.

### 6.5. Code Generation and Deployment

The final stage of ROOD methodology corresponds to the code generation. According to the scenario requirements, this code should be ready to be compiled in some of the platforms characterizing the different devices used in the scenario. Developers of the system will have to tackle this stage by implementing templates that filters the semantic information received from SOM models to transform it into text with sense for a specific compiler. In a canonical MDA approach, those templates are defined as Platform Specific Models (PSM); there will be as many different models as kinds of platforms are deployed on the scenario.

As stated in Section 6.2 (Table 7), the conceptualization process related to ROOD methodology considers two different platforms from a deployment point of view: those which have capability to interact with other devices (Native) and those which do not have capability to communicate each other and/or with other devices of the same platform (Gateways). Let us consider two devices defined in the scenario to illustrate the mechanisms for both cases:

- *The control module of the treadmill*: This control module is managed by a SunSPOT [61], which is a wireless embedded platform for managing sensors and actuators. Specifically, a SunSPOT node monitors and controls the mechanisms of the treadmill related to the working mode of the machine, actuating over it in case of receiving an alert from the *personal trainer*.
- *The smart T-shirt*: The platform of this device is provided by a Bioharness™ BT [62]. This is a non-intrusive platform enabling the capture of physiological data of users who wear it. The physiological data captured via this biometric belt is used to carry out quick diagnosis of the health status of the user.

The above-mentioned platforms are clear cases of the two kinds of platforms considered in ROOD methodology, Native and Gateway, respectively. The following paragraphs focus on describing on the specific development mechanisms for each one of them.

On one hand, the Sun SPOT controlling the treadmill mechanism is a programmable device with constrained resources; it is supplied by small battery (3.7 V rechargeable 770 mAh lithium-ion battery) and includes reduced memory (1 Mb of RAM and 8 Mb of Flash) and processor (400 MHz). Moreover, Sun SPOT nodes use specific communication protocols designed for embedded networks: IEEE 802.15.4 for radio and MAC layers and, in our case, 6LowPAN for network layer.

Sun SPOT can be considered as a *native* device in ROOD methodology. The development of software for Sun SPOTs has to be performed according to a reduced profile of Java Microedition (J2ME) and it uses a small Virtual Machine (VM) to execute bytecodes (Squawk). Applications (MIDlets in J2ME argot) deployed on Sun SPOTs have to follow the guidelines provided to develop software for the J2ME framework: those needs to extend the MIDlets class to allow the VM to manage the application lifecycle (create, start, pause, and destroy) during runtime via a concrete interface. Consequently, a developer, who needs to generate software artifacts for Sun SPOT, has to define a PSM that models the J2ME profile (at least the required part for the project) in order to filter the information coming from SOM model and transform it into code corresponding to MIDlets and auxiliary classes, implementing the required business logic. The specification of PSM is described in following section.

On the other hand, the Bioharness™ BT is an embedded device, which manages a set of biometric sensors. Its communication interface is based on Bluetooth and uses a proprietary application protocol to transmit frames with sensorial information to other devices that are listening for them. The Bioharness™ BT is considered as a *Gateway* device in ROOD methodology; it is not programmable, thus it does not natively support the deployment of software artifacts. We need an intermediary entity in order to deal with Bioharness™ BT that enables intercommunication with other devices and deploy necessary business logic to manage the gathered biometric information. As said in Section 1, we use an infrastructure entity to tackle this situation: the *Acquisition Platform*. This platform is based on a middleware build over a RESTlet framework that is executed in a device (server, PC, laptop, smart-phones, *etc.*) equipped with a variety of communication interfaces (Bluetooth, Wifi, Ethernet, Zigbee, *etc.*). The capabilities achieved through our *Acquisition Platform* are twofold: (i) to deal with a technologically heterogeneous ecosystem of non-programmable devices; (ii) to enable the deployment of RESTful services provided via software artifact generated with ROOD methodology. In our case study, the development of software for Bioharness™ BT depends on the *Acquisition Platform* whose logic is distributed in two parts: one deployed on the smart-phone of the user, and another on the gateway (see Figure 10). This entails the need of defining a PSM according to the RESTlet framework libraries in order to generate code for the *Acquisition Platform* both in smart-phone and gateway.

**Figure 15.**
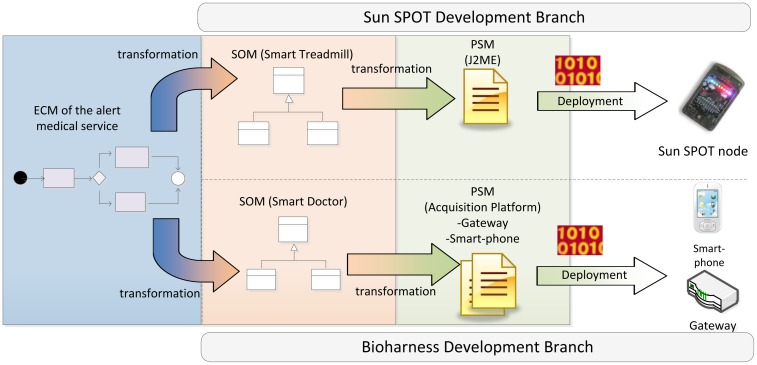
Development branches for the two cases considered following the ROOD methodology.

The two different cases of code generation explained in this section have to be conducted through two development branches (Figure 15). Both branches come from a common ECM model that generates two SOM models: (1) for Smart Treadmill corresponding with Sun SPOT platform; (2) for the Personal Trainer corresponding with a Bioharness™ BT, whose final generated code is deployed on the *Acquisition Platform* (parts corresponding to the gateway and the smart-phone of the user).

### 6.6. ROOD Implementation

This section gives some implementation details about the development of the MDA tool (so called *ROOD Visual Editor*) supporting the ROOD principles. The development of this tool is being tackled through different Eclipse projects [63]. The current version of this tool implements the ECM and SOM metamodels (viewpoints) and two modelers that enable a visual edition of the models defined in this work. Additionally, some transformation can be performed.

**Figure 16.**
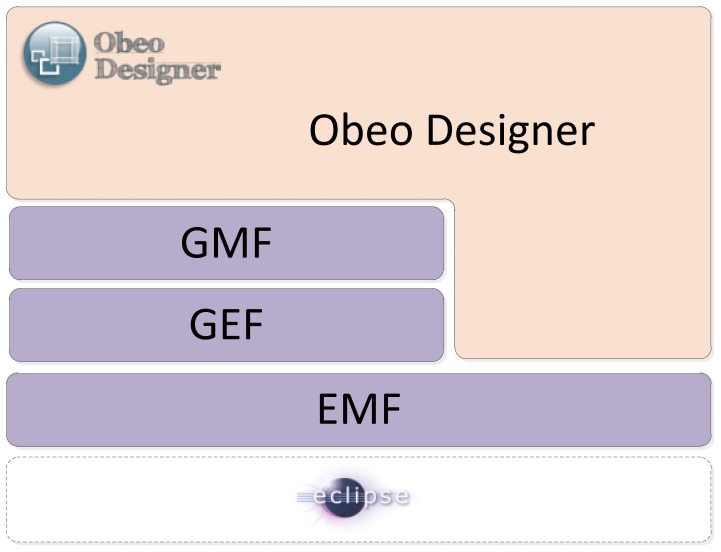
Obeo Designer architecture.

As said in previous sections, an important cornerstone of ROOD methodology is the Smart Space Modeling Language (SsML) that was designed as a UML profile. From that profile we designed two models, Environment Context Model (ECM) and Smart Object Model (SOM), which establish a well-defined Domain Specific Language (DSL) for modeling diverse aspects of smart spaces (activities, interactions, business logic and so on). Specifically, the Eclipse project provides the Eclipse Modelling Framework (EMF) [64] to support the development of DSLs based on its particular metamodelling language called Ecore. From Ecore files, EMF can generate a set of Eclipse plug-ins to edit, read, and serialize models according to the designed metamodels. However, the visual modeling through those plug-ins is only supported by treelike editors. Thus, additional configurations have to be performed in order to support a friendly visual modeling, for instance, using the Graphical Modelling Framework (GMF) [65]. Currently, there are other alternatives to EMF+GMF in order to facilitate the development of visual editors from metamodels. Particularly, we have used the Obeo Designer to develop our visual modeler for both ECM and SOM. Obeo Designer is based on the Eclipse Modeling frameworks (EMF, GEF and GMF) that provide tools to design and build modelers. Figure 16 illustrates the architecture of Obeo Designer.

Each one of those frameworks is very powerful by themselves. In contrast, they are characterized by their complexity to be understood and require a high level of expertise to build quality modeler. Obeo Designer integrates all those frameworks and hides such complexity. Using Obeo Designer, it is possible to define visual modelers without having knowledge about EMF and GMF technologies. The Ecore files are generated from “viewpoints” of the system that enable the creation of modelers (odesign files) by assigning specific graphical notation and behavior to every element composing the models.

**Figure 17.**
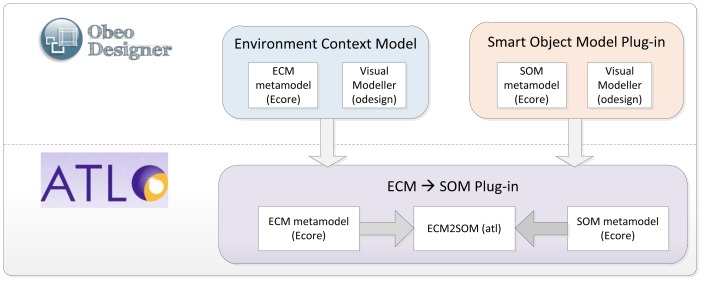
Eclipse plug-ins used for developing the ECM and SOM metamodels and their corresponding mapping to transform ECM into SOM.

Once we have created the Ecore and its corresponding modeler (odesign), we have to define mechanisms to automate the transformation between ECM and SOM through Model-to-Model (M2M) transformations. For this end, we use the ATL language [66] that is integrated in the Eclipse project. It provides an IDE that incorporates a set of tools (editors, debuggers, code completion, *etc.*) in order to conduct the coding of complex transformation. ATL manages metamodels defined in Ecore files to navigate and to edit them. Thus, ATL can take advantage from Ecore files generated by Obeo Designer, that we used to create the ECM and SOM metamodels and its modelers. This process is illustrated in Figure 17.

**Figure 18.**
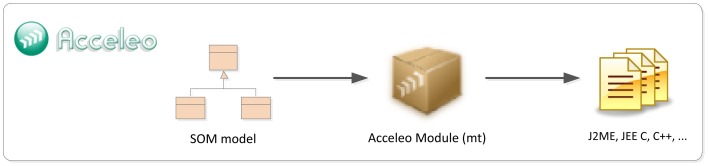
Code generation process using Acceleo.

Finally, we defined a mechanism to convert SOM models into code, that is, a Model-to-Text (M2T) transformation. For this end, we have chosen Acceleo [67], an Eclipse plug-in based on EMF to generate code. Acceleo provides a friendly development environment that allows creating code generators by means of a set of tools to lead the creation of transformation templates. Those templates are based on the Model to Test Language (MTL), an OMG's standard. Figure 18 shows the process to reach code from SOM model applying a specific Acceleo module that enables to achieve program code files defined in a programming language for a specific platform.

**Figure 19.**
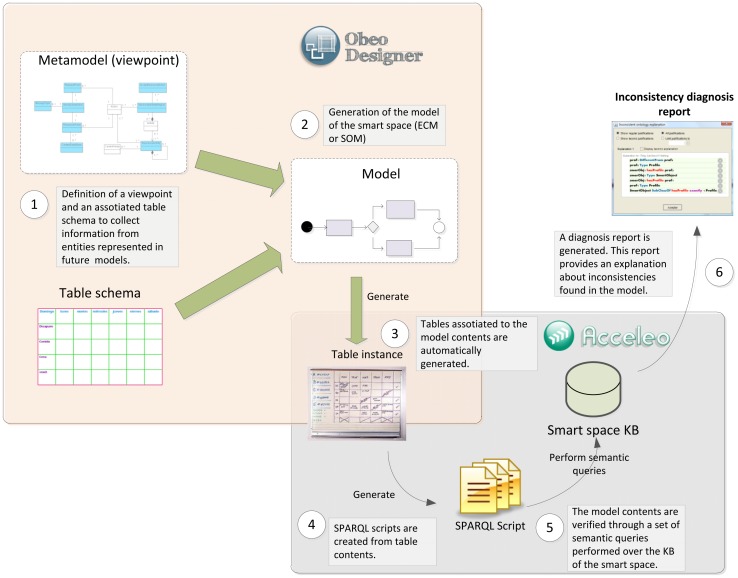
Verification process for validating the semantic information contained in a diagram.

To conclude the description of the *ROOD Visual Editor* implementation, we need to focus on the model verification issues. Through this mechanism we can verify consistency of the models according to the semantic information related to the smart space that is being modeled (e.g., sensors, actuators or tasks modeled for a specific smart object). We have taken advantage of the capability of Obeo Designer for tabulating the information represented in the models. The information collected in tables is then used to carry out corresponding verification process, as Figure 19 shows. In this way, elements and relationships used in ECM and SOM models are contrasted with the semantic information stored in the Knowledge Base related to the smart space that has to be modeled. This process is implemented by means of a set of semantic queries whose template is generated using Acceleo, which convert tables into SPARQL scripts. Those scripts are used to carry out recurrent semantic queries to Knowledge Base with the purpose of verifying the consistency of the models. Finally, a diagnosis report is generated and provided to model users (business engineer or software architect) with an explanation of the parts involved in the consistency. Thus, inconsistencies detected in models can be accordingly corrected.

Currently, this mechanism is performed off-line between each stage of the ROOD methodology, but it is expected that in subsequent versions of *ROOD Visual Editor* such functionality is integrated in the development environment as an Eclipse plug-in.

## 7. Conclusions

The design of a development methodology that provides common languages and procedures to tackle with a large heterogeneous smart space is currently an open challenge in the fields of the Internet of Things and, majorly, the Web of Things. The MDE approaches, specially the OMG standard, MDA, seem good solution to deal with a methodology aimed at this objective. MDA provides specific tools to construct development methodologies stratified in three conceptual layers (CIM, PIM and PSM levels). The MDA specification involves some important concepts that have to be taken into account in order to design optimized methodologies, among them the creation of modeling languages or the definition of accurate alignments between concepts in different layers. The final objective is to improve the quality of Model-to-Model (M2M) transformation to achieve a fine Model-to-Text (M2T) transformation, that is, the generation of program code that is compiled and deployed on hardware.

This work has demonstrated that it is possible to build Model-Driven methodologies based on MDA standards to tackle the challenges of smart spaces, *i.e.*, technological heterogeneity, variety of roles and reasoning, high ubiquity, and diversity of communication protocols, in order to achieve the rapid deployment of pervasive environments populated of smart objects seamlessly interconnected. We have proposed a Resource-Oriented and Ontology-Driven (ROOD) development methodology based on the principles of MDA to facilitate the development and deployment of smart spaces. Two kinds of models have been proposed in CIM and PIM levels (ECM and SOM, respectively) as well as some mapping rules to obtain the last from the former via transformations. Additionally, we have designed a verification process that use semantic technologies to verify consistency of models in relation to the semantic information of the smart space stored in a specialized Knowledge Base whose contents are shaped according to the Smart Object Ontology (SSO).

Currently, we are actively working on the development of the ROOD methodology with the support of open tools within the Eclipse project. This work includes, majorly, the definition of mappings rules to improve the transformation among ECM and SOM as well as the definition of templates to generate SPARQL script for semantic validation through Knowledge Bases. Further developments will try to integrate semantic validation mechanisms as an Eclipse plug-in within the ROOD Visual Editor supporting our methodology, which will implement several mechanisms for semantic debugging and model suggestion in order to support the modeling process during their creation. We are also planning to extend the SSO scope not only for describing deployment scenarios, but also to address its integration into the SsML metamodel in order to improve the verification of ECM and SOM models.

## Figures and Tables

**Table 1.** Comparative table for different approaches dealing with development aspects of smart spaces.

**Approach**	**Model-Driven mechanisms**	**Modeling constraints/ semantics**	**Standards**	**IoT/WoT mechanisms**	**User-centric programming**	**Modelling verification assistance**
Rauf *et al.* [32]	Yes (only for high level services. A DSL is provided)	Constraints from UML class and state diagram	HTTP, UML, WADL	Generates RESTful web services (limited for IoT deployments)	No	No
Christophe *et al.* [44]	Yes (to model many scenarios related to smart spaces)	Semantic descriptions (format not specified)	HTTP, H™L	Exposes object capabilities as RESTful services	Yes (only configurations at runtime)	Not specified
Simon *et al.* [45]	Yes	Constraints from canonical RESTful models	HTTP, XML, JSON	Generates software for RESTful servers for WoT	Yes (a toolkit is provided)	Partial (related to REST-based models)
Walter *et al.* [29]	Yes (MDA methodology to design DSLs is provided. DSL users can also take advantage of this approach)	Several mechanisms are implemented to verify consistency of metamodels and models	KM3, OWL, Ecore (metamodel), SPARQL	Not explicitly, but adaptable	A toolkit for designing DSLs and a Workbench to used those DSLs	Yes (Several verification and debugging mechanisms are provided (from metamodels and ontologies))
Katasonov *et al.* [16]	Yes (A MDE approach to model most of scenarios in relation to smart spaces)	Three ontologies to conduct modeling and transformations	OWL, UML, EMF	Not explicitly, but adaptable (generates code for smart spaces)	A toolkit for modeling smart spaces is provided	Yes (However, not specify that are the involved mechanisms)

**Table 2.** Graphic nodes included in SsML profile.

**Node Type**	**Notation**	**Description**	**Association and Constraints**
*AcceptSensorialAction*	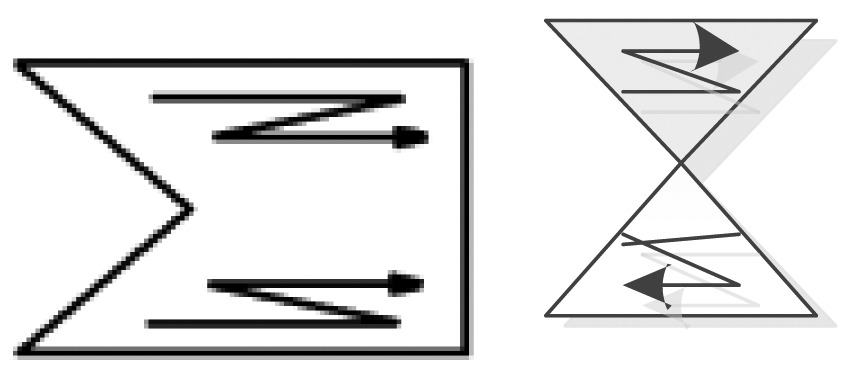	*AcceptSensorialAction* is an action that is performed when the occurrence of an event matches with a specified condition.	trigger:Trigger[1..*] The type of events accepted by the action, as specified by triggers. Only sensor, actuator and time events are allowed.
*ContextualDataStoreNode*	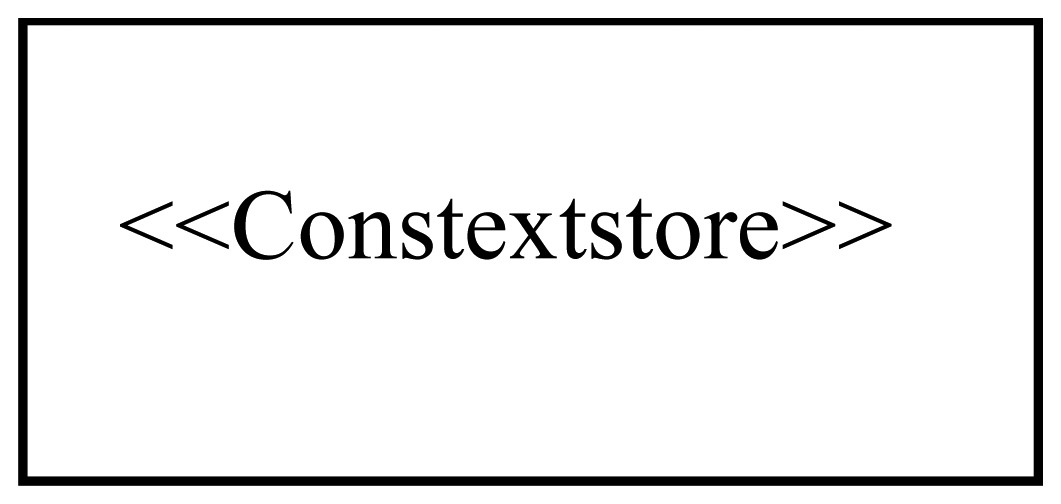	*ContextualDataStoreNode* is an element that allows storing contextual data of the smart space.	*InputPin:resourcePoint*[1] The input port through that context data are received. Only *ResourcePoint*s are allowed.
*SendQueryAction*	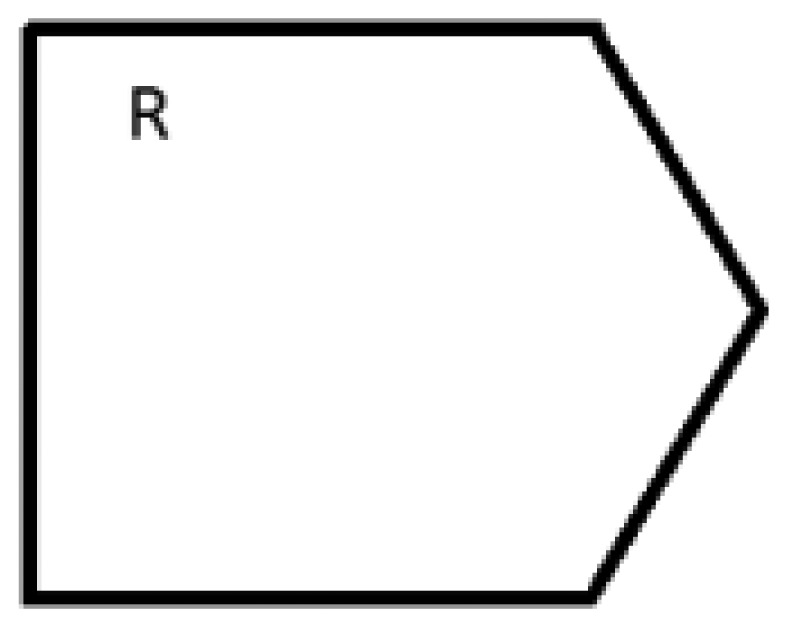	*SendQueryAction* creates a REST query from its inputs and transmit it to the target object, where it may cause a transition of a resource state or the execution of an activity.	*Query:restQuery*[1] The type of query transmitted to the target resource. Only REST queries are allowed. *Target:resourcePoint*[1] The target resource to which request is sent. Only *ResourcePoint*s are allowed. *Source:requestPoint*[1] The sender source for a request. Only *RequestPoint*s are allowed.

**Table 3.** Graphic paths included in SsML profile.

**Node Type**	**Notation**	**Description**	**Association and Constraints**
*RequestPoint*	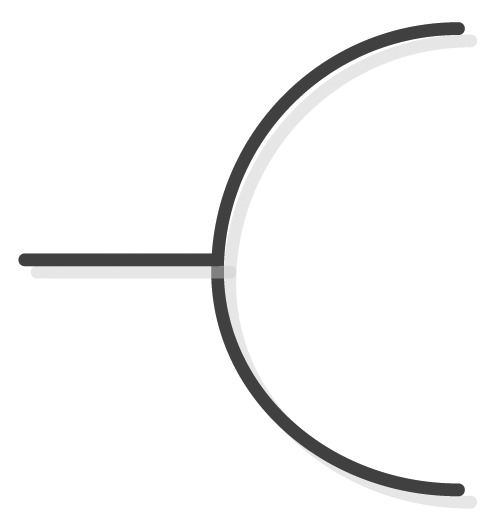	*RequestPoint* represents an output request for a resource related to an activity.	*outputValue:sendQueryAction*[1] Provides an output for a resource related to an activity. Only *SendQueryAction* inputs are allowed.
*ResourcePoint*	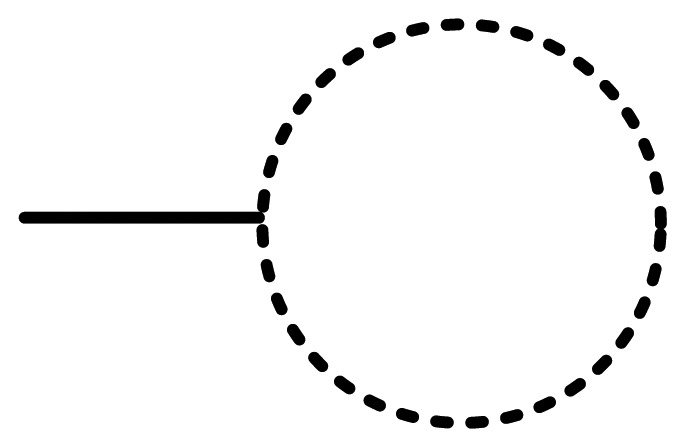	*ResourcePoint* represents a resource interface that triggers an action or activity in order to generate changes over a resource state.	*inputValue:sendQueryAction*[1] Provides an input for a resource related to an activity. Only *SendQueryAction*s are allowed.
*MessageType*	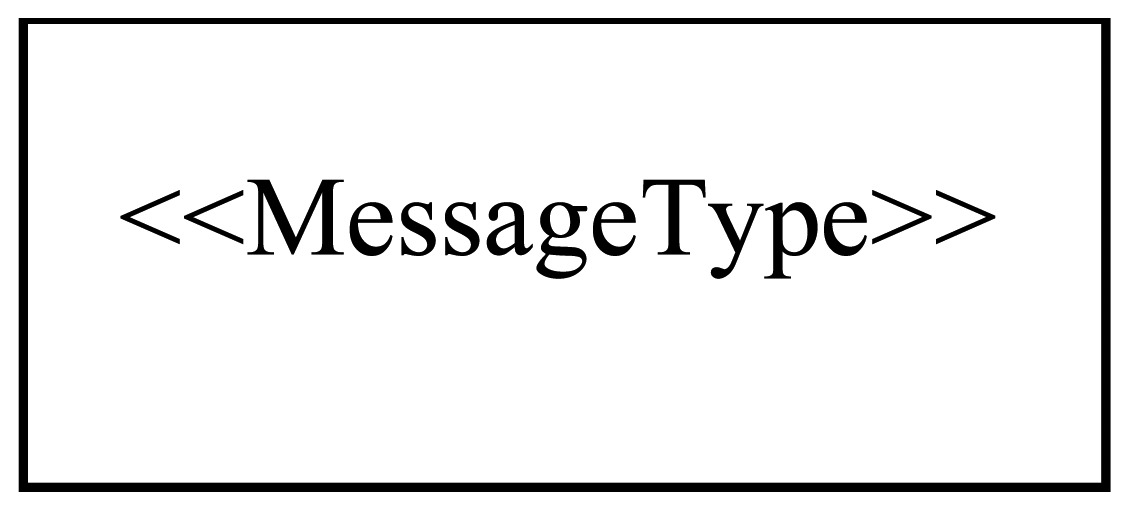	It represents the information exchanged between two actions. *MessageType* encapsulates the input, output and error messages based on protocols and/or architectures.	*Message:String[0..1]* It specifies the information encapsulated in the message payload. A *MessageType* must be *PrimitiveType*, *DataType* or another *MessageType*.

**Table 4.** Graphic elements for containment in SsML profile.

**Node Type**	**Notation**	**Description**	**Association and Constraints**
*ResourceActivity*	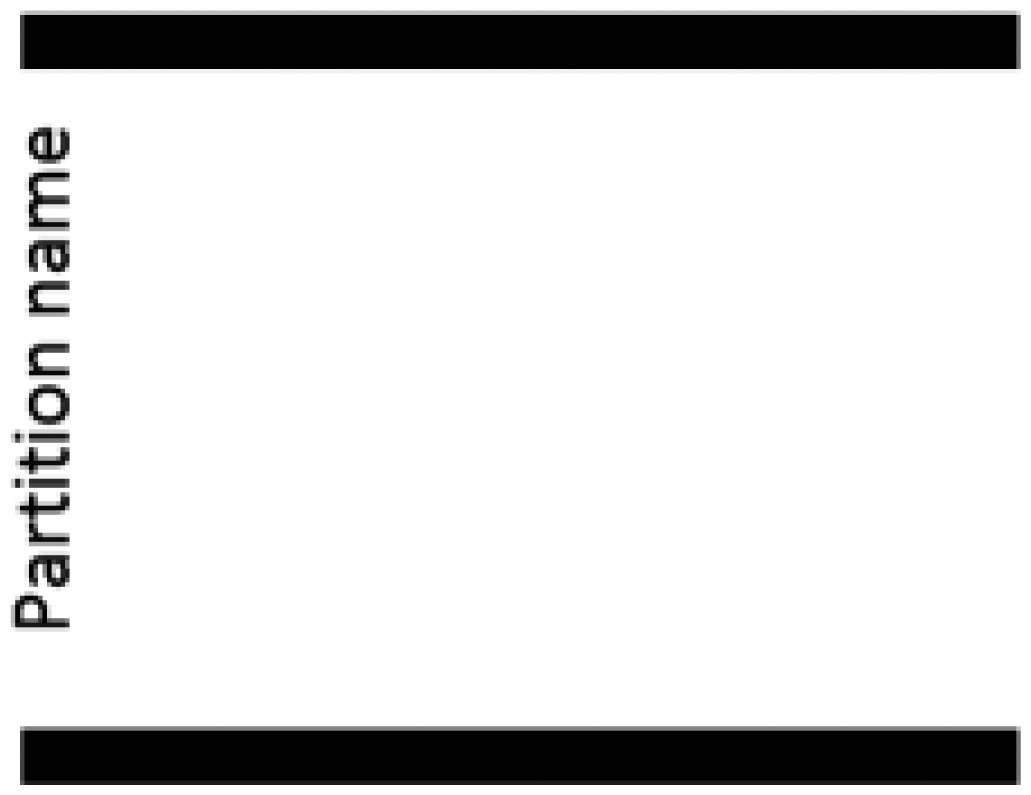	*ResourceActivities* are related to the intrinsic actions of resources. It defines partitions that define the organizational units in order to delimitate actions for specific resources in a deployment entity.	superPartition:ResourceActivity[0..1] Partition containing the partition. Only *ResourceActivity* partitions are allowed. subPartition:ResourceActivity[0..*] Partitions contained in the partition. Only *ResourceActivity* partitions are allowed.
*SensorialActivityRegion*	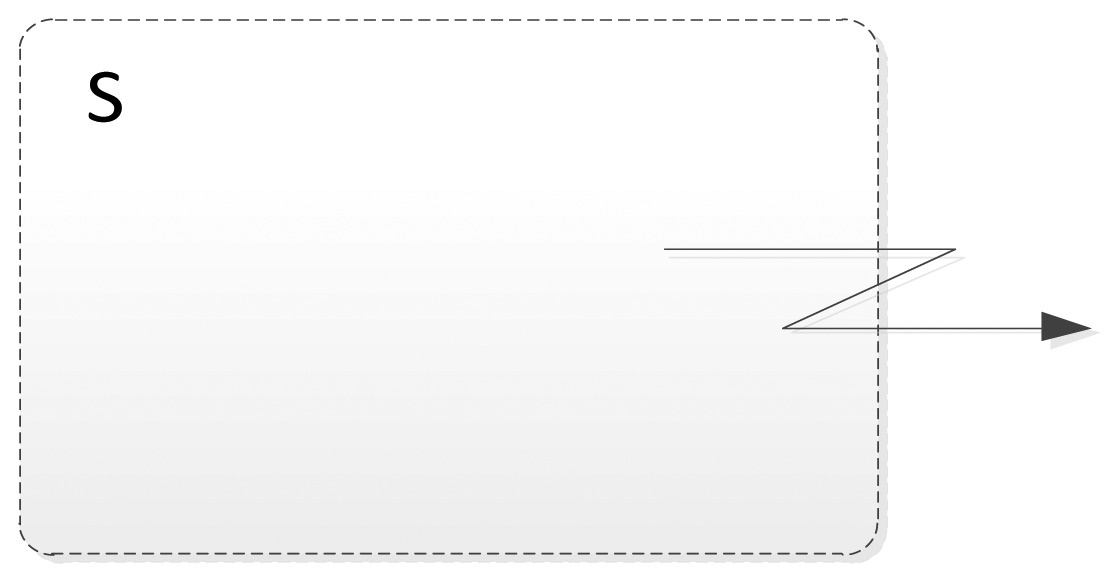	*SensorialActivityRegion* is an activity group that delimitates a set of action under the influence of a sensor work. When a sensorial event occurs into the *SensorialActivityRegion*, behaviours performing in the region are finished.	interruptingEdge:ActivityEdge[1..*] The boundaries leaving the region that will abort the activity being performed in the region. node:ActivityNode[0..*] Nodes contained in the region.

**Table 5.** Stereotypes of SsML defining concepts to be used in SOM.

**Stereotype**	**Description**
*Agent*	An *Agent* is a software entity that can adapt itself according to some environment parameters. Agent can acquire two roles: *Consumer* and *Provider*. The first one characterizes an agent that consumes information, and the second one characterizes an agent that is a source of information.
*Thing*	A *Thing* denotes any physical entity that acquires data through a set of agents working together in order to reach a common objective. In such a context a *Thing* can become a smart object as intended in this work. It is able to consume and provide resources following REST-based communication mechanisms.
*ThingArchitecture*	A *ThingArchitecture* is a community of *Things* that collaborate together in order to reach a common objective.
*Resource*	A *Resource* encapsulates and characterizes capabilities belonging to a *provider* and exposes them to be accessed and consumed by *consumers* using RESTful mechanisms.
*End-Point*	An *End-point* specifies an access point to a *resource*. It defines a method, address and message by means of which an agent can access and consume specific functionalities of the available resources.
*Expose*	An *Expose* dependency is used to describe capabilities and expose them through a document whose format is according to some standard.
*MessageType*	A *MessageType* represents the information exchanged between a resource and its consumer. It encapsulates input, output and error messages based on protocols and/or architectures. It is related to MessageType defined in ECM model.
*Process*	A *process* defines a workflow of tasks that are generally managed by an agent. Process behavior is not only influenced by agent interactions (both local and external) but also by real-world events gathered through sensors.
*Task*	A *task* is a procedure that is carried out at low level, e.g., sensorial information management or actuator behavior control.

**Table 6.** Traceability matrix indicating relationships between concepts in SSO and entities in ECM and SOM.

**Smart Space Ontology (SSO)**		**ECM viewpoint**	**SOM viewpoint**
Domain	Process	 Action, AcceptSensorialAction	 Task
	Sensor	 ResourceActivity	✖
	Actuator	 ResourceActivity	✖
	Sensing	 SensorialActivityRegion	 Task
	Actuating	 Action	 Task
	SmartObject	 ResourceActivity	 SmartObject
Service	Consumer	 RequestPoint	 Consumer
	Provider	 ResourcePoint	 Provider
	Process	 Action, ResourceActivity	 BusinessProcess
	Task	 Action	 Task
Resource	Method	SendQueryAction	 RESTInterface
	URI	✖	 RESTInterface
	MessageInput	 MessageType	 Message
	MessageOuput	 MessageType	 Message

**Table 7.** Report of the analysis of domain aspects in the smart gym.

**Smart Space**	**Smart Object**	**Platform** ^1^	**Device**	**Sensor**	**Actuator**
Smart Gym*	Personal Trainer	Native	Smartphone	✖	Touch screen

		Gateway	Smart T-shirt	Heart monitor (Sensing heart rate)	✖

				Body temp. sensor (Sensing body temp.)	
				Accelerometer (Monitoring body movement)	

	Smart Treadmill	Native	Embedded actuator device	✖	Screen

					Treadmill mechanisms

	Client Contextualizer	Native	Server	✖	✖

	Emergency Manager	Native	Smartphone		✖

(*) This table only contains information related to the alert medical service, which belongs to the smart gym.

1 SSO considers two kinds of platforms: (a) Native (generated artifacts are natively deployed on the device); (b) Gateway (generated artifacts are deployed on a Gateway in order to reach those devices that do not have its own communication capabilities).

**Table 8.** Report of the analysis of service aspects in the smart gym.

**Smart Object**	**Participant**	**Role (consumer/ provider)**	**Business Process**	**Input**	**Output**	**Task**
Personal trainer	Health monitor	Provider	Heart sensing	Configuration data	Heart rate	Sensing heart rate

			Body temp sensing	Configuration data	Body temp.	Sensing body temp.

	Activity monitor	Provider	Activity sensing	Configuration data	Activity description	Sensing activity

	Alert generator	Consumer/ provider	Heart monitoring	Heart rate	✖	✖

			Body temp monitoring	Body temperature	✖	

			Activity monitoring	Activity description	✖	

			Health analyzing	Vital signs (heart/body temp.) and activity.	Alert (health/fall) notification	

Smart Treadmill	Treadmill manager	Consumer	Mechanism manager	Configuration data	Messages (e.g., alert message, work progress)	Treadmill config.
	
			Message manager	Alert notification		Showing messages on screen
Client Contextualizer	Selector	Provider	Obtaining user data	✖	User health profile	✖

	Inserter	Consumer	Inserting user data	User health report	✖	✖

Emergency Manager	Alert handler	Consumer	Message managing	Emergency message	✖	Showing messages on screen

**Table 9.** Report of the analysis of resource aspect in the smart gym.

**Smart Object**	**Participant**	**Resource**	**Operation**	**HTTP method**	**Input message**	**Output message**
Smart Treadmill	Treadmill manager	Alert manager	Set alert messages	PUT	JSON{“alert_type“:”[mode]”,“working_mode”:”[mode]”,“act_recom”:”[suggest]”}	✖

			Obtain working mode	GET	✖	JSON:{“working_mode”:”[“mode”,”speed”]}

Client Contextualizer	Selector	Data base	Obtain user profile	GET	JSON{“query“:{“type”:”select”,“body” :[{”user_id”:”[id]”},{“req”: “health_cond”},{“req” : “illnesses”} ] }}	JSON{“illnesses”:[list of illnesses],“health_cond”:[list of health conditions]}
	
	Inserter		Update health report	PUT	JSON{“query“:{“type”:”insert”,body :[{”user_id”:”[id]”},{“heart_rate”: “[rate]”},{“body_temp” : “[temp]”} ] }}	✖

Emergency Manager	Alert handler	Emergency manager	Notify an emergency situation	PUT	JSON{“notification“:”[type]”,“message”: “[text]” }	✖
